# Integrating Pretreatment Circulating Tumor HPV DNA and Tumor Volume for Risk Stratification in HPV-Positive Oropharyngeal Squamous Cell Carcinoma

**DOI:** 10.3390/cancers18142194

**Published:** 2026-07-08

**Authors:** Lin Zhu, Chunying Shen, Wei Qian, Peiyao Liu, Tingting Xu, Huijuan Liu, Xin Zhou, Xueguan Lu

**Affiliations:** 1 Department of Radiation Oncology, Fudan University Shanghai Cancer Center, 270 Dong’an Road, Shanghai 200032, China; zhulin_@fudan.edu.cn (L.Z.); shency2016@163.com (C.S.); qianwei2005ahmu@163.com (W.Q.); nancyalec@sina.com (P.L.); dr_tingtingxu@163.com (T.X.); 15110972653@163.com (H.L.); 2 Department of Oncology, Shanghai Medical College, Fudan University, Shanghai 200032, China; 3 Shanghai Clinical Research Center for Radiation Oncology, Shanghai 200032, China; 4 Shanghai Key Laboratory of Radiation Oncology, Shanghai 200032, China

**Keywords:** HPV-positive oropharyngeal squamous cell carcinoma (OPSCC), circulating tumor HPV DNA, liquid biopsy, tumor burden, risk stratification

## Abstract

Background: Circulating tumor HPV DNA (ctHPV DNA) has shown clinical value in HPV-positive oropharyngeal squamous cell carcinoma (OPSCC), but its relationship with tumor burden and its role in prognosis, especially when combined with clinicoradiological factors, remain to be further defined. Methods: We analyzed 103 patients with HPV-positive OPSCC enrolled in an observational biomarker study, most of whom received induction chemotherapy or chemoimmunotherapy followed by definitive radiotherapy. Blood samples were collected at pretreatment, post-radiotherapy, and follow-up time points, and ctHPV DNA levels were quantified using droplet digital PCR. Baseline clinicoradiological parameters from contrast-enhanced MR or CT and ^18^F-FDG PET/CT were analyzed for their association with ctHPV DNA levels and clinical outcomes. Results: Baseline ctHPV DNA correlated with primary tumor volume (V_T_), nodal volume (V_N_), total tumor volume (V_T+N_), and the number of involved primary tumor sites and lymph node regions, particularly LN-related features. V_T_ and maximum LN diameter were independent predictors of baseline ctHPV DNA. Detectable follow-up ctHPV DNA was associated with inferior progression-free survival but showed low sensitivity and positive predictive value for relapse detection. Integrative stratification using baseline ctHPV DNA and V_T+N_ identified a potential high-risk subgroup with high tumor volume but paradoxically low ctHPV DNA (VOL^high^DNA^low^). Exploratory RNA-seq analysis was performed to investigate potential biological features underlying this discordance, and a seven-gene signature associated with PFS was identified. Conclusions: These findings suggest that baseline ctHPV DNA reflects tumor burden and may provide additional information for upfront risk stratification in HPV-positive OPSCC, warranting further validation in larger prospective cohorts.

## Simple Summary

Some patients with HPV-positive oropharyngeal cancer still develop recurrence after treatment, and better tools are needed to identify high-risk patients early. This study evaluated circulating tumor HPV DNA in patients treated with induction therapy combined with definitive radiotherapy. Pretreatment circulating tumor HPV DNA was closely associated with tumor burden, especially lymph-node-related tumor volume. Follow-up circulating tumor HPV DNA detected by our droplet digital PCR assay showed limited sensitivity for predicting recurrence in this cohort. By combining pretreatment circulating tumor HPV DNA with total tumor volume, we identified a potential high-risk subgroup with a large tumor burden but unexpectedly low HPV DNA levels, suggesting that blood-based testing and imaging-based tumor volume may provide complementary information for risk stratification.

## 1. Introduction

The global incidence of HPV-positive oropharyngeal squamous cell carcinoma (OPSCC) has been rapidly rising over the last few decades [1]. As a distinct entity, HPV(+) OPSCC exhibits higher radiosensitivity [2] and superior prognosis [3] when compared with its HPV-negative counterpart. This discrepancy has fueled growing interest in treatment de-escalation for HPV(+) OPSCC [4]. However, despite encouraging results from some phase II trials, randomized trials have failed to demonstrate the non-inferiority of de-escalated treatment even after ruling out patients with high-risk features (T4, N3, or smoking history > 10 pack-years) [5,6]. These findings suggest a limited value for conventional clinical parameters in patient selection, highlighting an unmet need for a more precise biomarker to improve risk stratification in HPV(+) OPSCC.

Circulating tumor HPV DNA (ctHPV DNA) has emerged as a novel liquid biopsy biomarker in HPV-related malignancies. Accumulating evidence has supported the clinical utility of ctHPV DNA in early diagnosis, response evaluation and posttreatment surveillance in HPV(+) OPSCC [7]. However, prognostication using ctHPV DNA has been challenging. Although follow-up ctHPV DNA has been widely acknowledged as a prognostic indicator [8,9], its delayed timing lends itself more to salvage therapy upon recurrence than to upfront risk stratification. Earlier time points, including baseline [10], post-induction [11] and postoperative [12] ctHPV DNA, have yielded controversial results regarding their predictive value, limiting their applicability for timely treatment modification. Moreover, although ctHPV DNA has been reported to reflect tumor burden [13], it alone may be insufficient to yield adequate prognostic precision given the complex interplay with clinicopathologic factors [14,15]. While evidence from other cancers has shown that integrative models combining clinical and ctDNA biomarkers improve prognostic efficacy [15,16], their role in HPV(+) OPSCC remains unexplored.

Induction chemotherapy has been investigated as a response-adaptive strategy in selected patients with HPV-associated OPSCC, allowing for subsequent treatment de-intensification or escalation according to early tumor response [17,18]. Recent evidence suggests that rapid early clearance of ctHPV DNA during neoadjuvant therapy may serve as a promising biomarker for treatment response and risk stratification. More recently, immune checkpoint inhibitors have also been incorporated into induction or perioperative treatment strategies for head and neck squamous cell carcinoma, including HPV-positive OPSCC [19]. However, the clinical utility of ctHPV DNA monitoring in patients receiving induction-based therapy followed by definitive radiotherapy remains insufficiently defined. Therefore, ctHPV DNA dynamics in this treatment context require specific evaluation.

In this study, using an HPV(+) OPSCC cohort treated with definitive radiotherapy (RT), we analyzed the correlation between ctHPV DNA and clinicoradiological tumor features, aiming to identify independent determinants of pretreatment ctHPV DNA levels. Furthermore, the prognostic efficacy of ctHPV DNA was evaluated at different time points to explore the possibility of advancing ctHPV DNA-guided risk stratification to the pretreatment setting, with a particular focus on the feasibility of integrative prognostication via clinic-biological integration in HPV(+) OPSCC.

## 2. Methods and Materials

### 2.1. Study Design and Eligibility

The study cohort comprised patients with HPV(+) OPSCC from a single-institutional observational biomarker study. The patients received induction chemotherapy/chemoimmunotherapy followed by radiotherapy. Eligible patients included in this study all had accessible pretreatment radiological images for qualitative and quantitative evaluation, including computerized tomography (CT), magnetic resonance imaging (MRI) or ^18^F-fluorodeoxyglucose positron emission tomography (^18^FDG-PET-CT). The regions of interest (ROIs) were manually delineated and reviewed independently by two experienced radiologists. All ROIs of the primary tumor and LN were segmented on CE-T1WI and T2WI individually in a slice-by-slice manner throughout the whole tumor, including all parenchymal and necrotic areas. Neck contrast-enhanced CT was also used to cross-check metastatic lymph-node volume and reduce measurement bias. HPV positivity was confirmed by tissue-based assessment. The p16 immunohistochemistry (IHC) positivity was defined as positive staining in more than 70% of tumor cells. Tissue HPV genotyping was primarily performed using PCR-reverse dot blot (PCR-RDB) hybridization (99 patients). The PCR-RDB assay covered the following HPV genotypes: HPV16, 18, 33, 35, 39, 45, 51, 52, 53, 56, 58, 59, 66, 68, 73, 83, 82, 6, 11, 42, 43, and 81. Four patients were confirmed by RNA-seq-based HPV detection using tumor tissue samples. Clinical characteristics, including tumor stage according to the 8th (TNM8) and 9th (TNM9)-edition staging systems of the American Joint Committee on Cancer (AJCC), smoking history, and treatment modalities, were collected from medical records.

### 2.2. Surveillance Plan

This comprises serial clinical follow-up visits with physical examinations and restaging imaging approximately 3 months after treatment completion, with additional imaging at the clinicians’ discretion. In this study, the ctHPV DNA surveillance plan referred to longitudinal blood-based monitoring from baseline through the posttreatment follow-up period. In addition to usual surveillance, ctHPV DNA testing was performed for subjects at prespecified intervals posttreatment during surveillance, generally corresponding to surveillance follow-up visits, including: before treatment (baseline); 0–4 weeks after radiation completion; 3 months after treatment; every 3 months up to 2 years after treatment. Subjects with at least one surveillance ctHPV DNA test were included in the final analysis. Medical record abstraction was performed to ascertain pertinent clinical characteristics. The date of progression (recurrence or metastasis) was considered the date of tissue diagnosis when available, or otherwise the date of imaging showing highly suspicious findings consistent with presumed progression. Persistent disease is coded as recurrence in this analysis.

### 2.3. Blood Sample Collection and ctHPV DNA Analysis

Blood samples were collected for ctHPV DNA analysis at prespecified time points, including baseline, 0–4 weeks after completion of RT, 3 months after treatment and every 3 months thereafter for up to 2 years. Subjects with at least one surveillance ctHPV DNA test were included in the final analysis. Peripheral blood (12 mL) was collected in K2E (EDTA) tubes, and plasma was separated within 1 h by double centrifugation at 1600× *g* and 16,000× *g* at 4 °C for 10 min each. Cell-free DNA was extracted from 2 mL of plasma using a column-based kit (Magen, Guangzhou, China). ctHPV DNA was quantified by droplet digital PCR (ddPCR) targeting HPV16, HPV18, and HPV33, with GAPDH as an internal control. Droplets were generated using the QX200 system (Bio-Rad Laboratories, Hercules, CA, USA), amplified on a T100 PCR instrument (Bio-Rad Laboratories, Hercules, CA, USA), and analyzed on the QX600 platform (Bio-Rad Laboratories, Hercules, CA, USA). ctHPV DNA levels were calculated as copies/mL plasma and expressed as log10 HPV DNA copies/mL. The plasma ctHPV DNA assay covered HPV16, HPV18, and HPV33, and detailed methods and primer/probe information are provided in the Supplementary Methods.

### 2.4. Assessment of Clinicoradiological Parameters

Oncologic features were collected on MR or CT images, including individual anatomical structures involved by the primary tumor, the number of involved structures, lymph nodes (LNs) laterality, retropharyngeal LN metastasis, LN necrosis, imaging extranodal extension (iENE), the number of metastatic LNs, the number of lymphatic drainage areas, and maximum lymph node diameter (MLD). The quantitative volume of primary tumor (V_T_) and metastatic LNs (V_N_) was measured independently via reconstruction of regions of interest (ROI) per slice on contrast-enhanced MR or CT images. The metabolic activity of primary tumors and LNs was measured on PET-CT via maximum normalized uptake (SUVmax) of primary tumors (SUVmax-T) and LNs (SUVmax-N) respectively.

### 2.5. Bulk RNA-Sequencing

Total RNA from FFPE samples was isolated by using the RNAstorm FFPE RNA Isolation Kit (Cell Data Sciences, Fremont, CA, USA). To eliminate DNA contamination, total RNAs were treated with DNase I (New England Biolabs, Ipswich, MA, USA). The strand-specific RNA-seq libraries were prepared by using the SMARTer Stranded Total RNA-Seq Kit—Pico Input Mammalian (Clontech Laboratories, Mountain View, CA, USA). Quality control was performed using Qubit (Thermo Fisher Scientific, Waltham, MA, USA) and Qsep100 (BiOptic, Taiwan, China) before the libraries were sequenced on the Illumina NovaSeq platform (Illumina, San Diego, CA, USA) using a 150 bp paired-end run. The RNA-seq data sequencing reads were aligned to the reference genome (Genome Reference Consortium GRCh38) using the spliced read aligner STAR, which was provided with the Ensembl human genome assembly. The gene expression matrix was obtained by featureCounts and normalized by FPKM for downstream analysis.

### 2.6. Construction of Multi-Gene Prognostic Signature

Multivariable linear regression was performed to screen genes significantly correlated with baseline ctHPV DNA levels. Univariable Cox proportional hazards analysis was used to identify genes associated with progression-free survival (PFS). Genes significant in both analyses were intersected and further refined using LASSO Cox regression. A risk score was then constructed using a ridge-penalized Cox model (alpha = 0), with the optimal penalty parameter (lambda) selected by 5-fold cross-validation. The cutoff value was determined using maximally selected rank statistics. Model performance was assessed by Harrell’s C-index. Internal validation was conducted using bootstrap resampling (1000 iterations) to estimate optimism-corrected performance and leave-one-out cross-validation to assess sensitivity to individual observations. Model accuracy and clinical utility were further evaluated using bootstrap-corrected calibration plots, time-dependent ROC curves at 1 and 2 years, and decision curve analysis.

### 2.7. Statistical Analysis

Categorical variables (CATs) were summarized as frequencies and percentages, and continuous variables were reported as median (IQR) and mean ± SD, as appropriate. Normality was assessed using the Shapiro–Wilk test. Group comparisons were performed using the t-test, Wilcoxon rank-sum test, or Kruskal–Wallis test, as appropriate. Correlations between ctHPV DNA and clinical variables were evaluated using Pearson, Spearman, or point-biserial correlation coefficients, with bootstrap resampling used to estimate 95% confidence intervals when applicable. Univariable and multivariable linear regression models were fitted to identify predictors of baseline ctHPV DNA level, with stepwise selection based on the Akaike information criterion. Survival curves were estimated by the Kaplan–Meier method and compared using the log-rank test. Cox proportional hazards models, including interaction models when appropriate, were used to estimate hazard ratios and assess prognostic associations. Model performance was evaluated using Harrell’s C-index and likelihood ratio tests. Multiple testing was controlled using the Benjamini–Hochberg method, with Bonferroni correction also reported where applicable. All analyses were performed in R version 4.4.2. All tests were two-sided, and *p* < 0.05 was considered statistically significant.

## 3. Results

### 3.1. Patient Characteristics

A total of 103 patients were included in this study, with a median age of 55 years. The HPV status was confirmed with dual tests of p16 and HPV genotypes in 95.2% of patients. In total, 40.8% of patients were nonsmokers. According to TNM9, 38.8% and 82.5% of patients had T3–4 and N2–3 disease, respectively. Radical RT-based treatment was planned for all patients. Induction chemotherapy with or without immunotherapy was given to 90.3% of patients, and 99 patients (96.1%) completed the planned RT course. Among the 103 patients with available baseline ctHPV DNA data, 83 had detectable ctHPV DNA, yielding a detection rate of 80.58%. Detailed patient characteristics are summarized in Table 1.

### 3.2. Baseline ctHPV DNA Level Correlates with Clinicoradiological Tumor Characteristics

**Figure 1:**
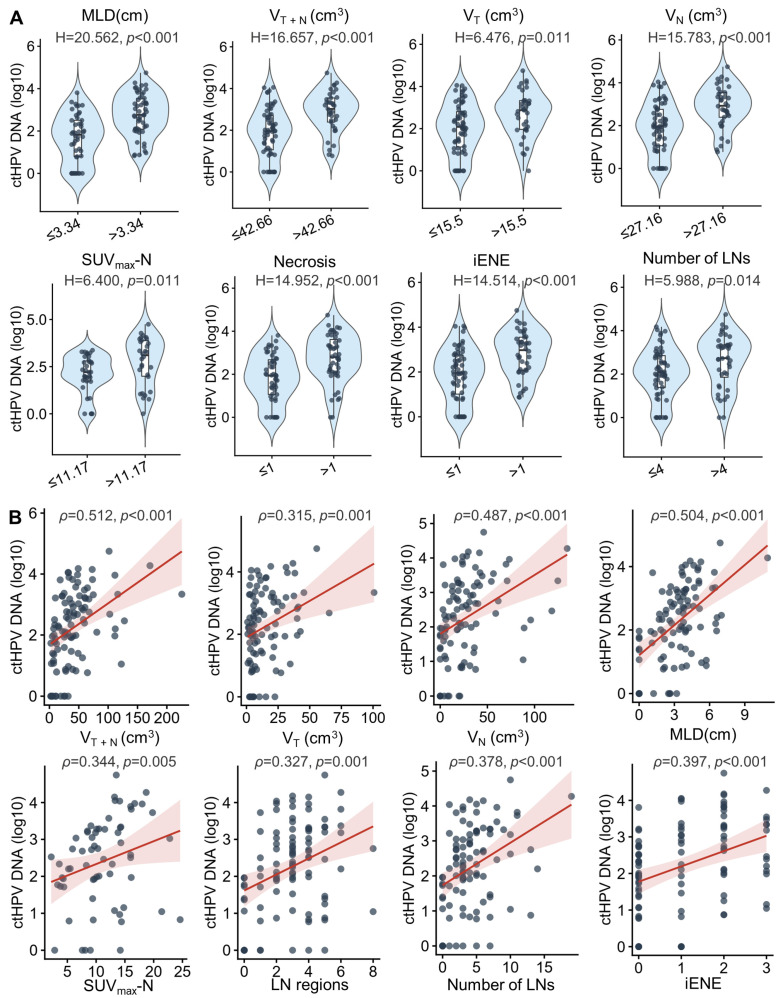
Correlation of baseline ctHPV DNA level with clinical factors. (**A**) Violin plots presenting distributions of categorical clinical features with ctHPV DNA. Kruskal–Wallis H tests were performed to assess differences in ctHPV DNA (log10) distributions between groups; H statistics and *p*-values (scientific notation) are annotated in each panel. Samples were grouped according to ctHPV DNA baseline levels above and below the median. (**B**) Scatter plots of correlations between ctHPV DNA and tumor characteristics. All correlations were assessed using Spearman’s rank correlation test (non-parametric). Bootstrap resampling was performed to calculate 95% percentile confidence intervals (CIs) for Spearman’s rho (*ρ*). Linear regression lines (solid lines) with 95% confidence interval ribbons (shaded areas) are overlaid to visualize the trend of associations. Statistical significance was adjusted for multiple comparisons using the false discovery rate (FDR) and Bonferroni methods. V_T_: volume of primary tumor; V_N_: volume of metastatic nodes; V_T+N_: total tumor volume; LN: metastatic lymph nodes; SUV_max_-N: maximum standardized uptake value (SUV_max_) of lymph node; MLD: maximal lymph node diameter; ENE: extranodal extension.

The primary tumor and metastatic nodes had a median SUVmax of 13.3 and 11.2, respectively. Median V_T_, V_N_ and V_T+N_ values were 15.5, 27.2 and 42.7 cm^3^, respectively. Metastatic nodes had a median MLD of 3.34 cm; radiological interpretation showed that 62.6% of patients had iENE and 60.6% had central necrosis, including 19.2% with cystic lesions (Table S1). Through converting continuous variables into categorical features using the median value as the cutoff, intergroup comparison showed a significantly higher ctHPV DNA (log10 transformed) in subgroups with smoking history, higher T/N classification and overall stage, larger tumor volume (V_T_, V_N_, V_T+N_) and quantitative structures being involved (number of primary tumor-involved structures, number of metastatic LN regions) (Figure S1). Specifically, ctHPV DNA showed more significant differences in extensive LN-related features, including N classification, V_N_, MLD, SUVmax-N, laterality, cystic/necrotic status and iENE (Table S2, Figure 1A), while T-related variables such as T classification, SUV_max_-T and individual involved structures were less correlated (Table S3).

Correlation analysis followed by a univariable linear regression demonstrated a strong linear correlation between baseline ctHPV DNA, V_T+N,_ V_T,_ and stage, as well as quantitative LN features, including iENE, necrosis, number of metastatic LNs and regions, MLD, V_N_, and SUVmax-N (Tables S4 and S5; Figure 1B and Figure S2). Using a composite score derived from the significance of intergroup differences, ctHPV DNA-to-clinical correlation and linear fitting (Table S6), variables were selected for a multivariable linear regression model. As a result, V_T_ and MLD were retained as independent predictors of baseline ctHPV DNA load (Table S7).

### 3.3. Follow-Up ctHPV DNA Detectability Predicts Survival Outcomes

**Figure 2:**
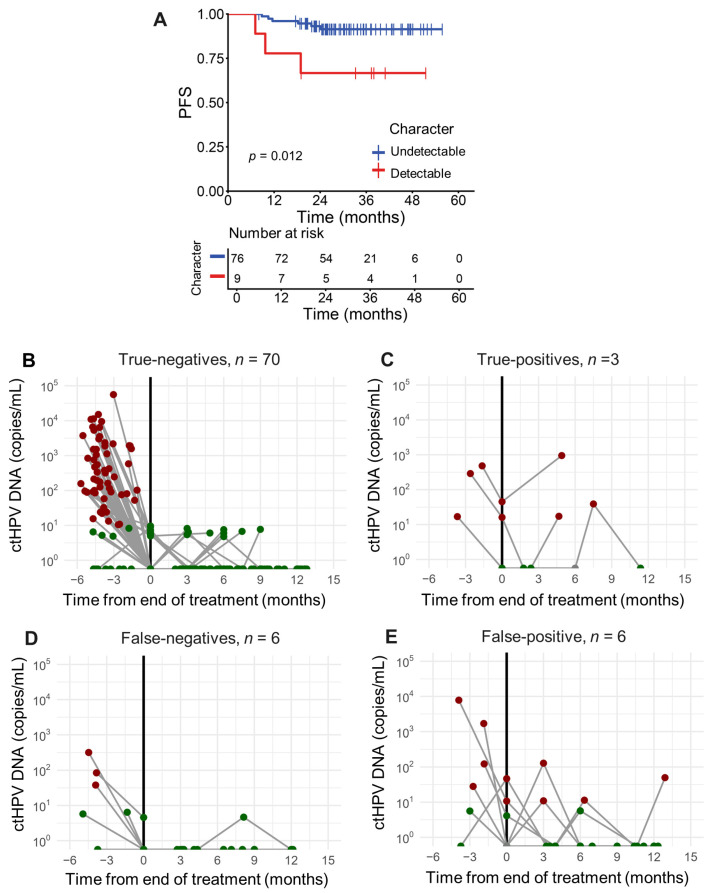
Correlation of posttreatment ctHPV DNA with survival outcomes. (**A**) Progression-free survival (PFS) among patients with and without detectable ctHPV DNA during posttreatment follow-up for HPV-positive oropharyngeal squamous cell carcinoma. Log rank *p* = 0.012; HR, 5.19 (95% CI, 1.24–21.73). (**B**–**E**) ctHPV DNA trajectories before and after treatment in HPV-positive oropharyngeal squamous cell carcinoma. (**B**) True negatives (*n* = 70): undetectable ctHPV DNA with no evidence of disease at all posttreatment time points. (**C**) True positives (*n* = 3): disease progression with detectable ctHPV DNA during follow-up. (**D**) False negatives (*n* = 6): undetectable ctHPV DNA at the time of recurrence diagnosis. (**E**) False positives (*n* = 6): detectable ctHPV DNA posttreatment with no evidence of disease at last follow-up. All samples with follow-up test results were included. Each line represents one patient. Time 0 (vertical solid black line) denotes the end of treatment. Red dots indicate detectable ctHPV DNA, and green dots indicate undetectable ctHPV DNA.

After completion of definitive treatment, ctHPV DNA results were available for 85 patients during follow-up. The follow-up ctHPV DNA detectability showed a significant correlation with treatment outcomes in survival analysis. Patients with detectable follow-up ctHPV DNA had significantly inferior 2-year PFS (94.0% vs. 68.6%) (Figure 2A). Cox proportional hazards analysis showed that detectable follow-up ctHPV DNA was associated with an approximately fivefold increased risk of progression or death (HR, 5.19; 95% CI, 1.24–21.73; Wald *p* = 0.024).

Furthermore, tumor relapse occurred in 9 of 85 patients (10.6%), including six with local and/or regional recurrence, two with distant metastasis alone, and one with both regional recurrence and distant metastasis. Of these patients, two underwent successful salvage surgery, four remained alive with disease after chemoimmunotherapy, and three ultimately died. Follow-up ctHPV DNA was detectable in nine patients, of whom three developed clinical relapse, with lead times of 0, 29, and 39 days. Among the 76 patients with persistently undetectable ctHPV DNA, six developed relapse (7.9%); notably, five of these relapses (83.3%) occurred within irradiated locoregional regions. The sensitivity, specificity, positive predictive value (PPV), and negative predictive value (NPV) of follow-up ctHPV DNA for tumor progression were 33.3%, 92.1%, 33.3%, and 92.1%, respectively (Table S8, Figure 2B–E).

### 3.4. Tumor Volume–ctHPV DNA Discordance Identifies a High-Risk Subset (VOL^high^DNA^low^) of HPV(+) OPSCC

**Figure 3:**
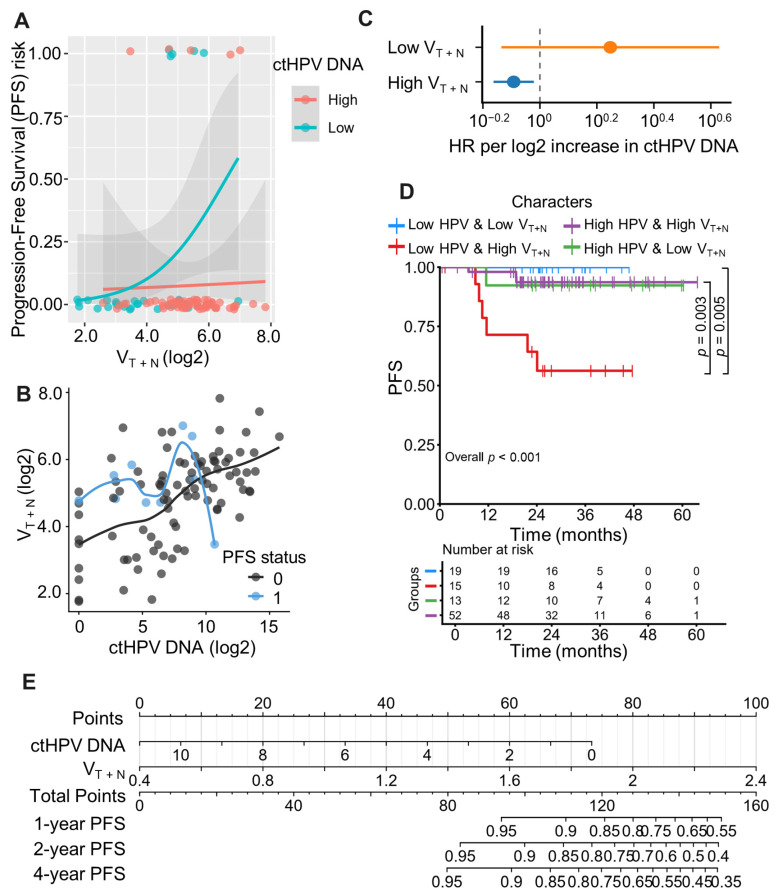
Interaction between ctHPV DNA and tumor burden determines progression risk. (**A**) Continuous Cox interaction model evaluating the interaction between baseline ctHPV DNA and tumor burden (V_T+N_) for PFS. (**B**) Two-dimensional risk heatmap showing the distribution of ctHPV DNA and tumor burden stratified by progression status. (**C**) Forest plot of stratified Cox models demonstrating effect modification. The dashed vertical line indicates HR = 1 (10^0^). (**D**) Kaplan–Meier curves of four biologically defined groups based on ctHPV DNA and tumor burden, using PFS as the endpoint. (**E**) Nomogram for predicting the probability of PFS in patients. The model integrates two independent prognostic factors: ctHPV DNA and tumor burden (V_T+N_). C-index = 0.720; bootstrap-corrected C-index = 0.697. All ctHPV DNA and V_T+N_ values shown in the figure were log2-transformed. *p*-values in (**D**) were calculated using the log-rank test. ctHPV DNA was dichotomized at 85 copies/mL: values < 85 copies/mL were classified as “low,” and values ≥ 85 copies/mL were classified as “high.” V_T+N_ was dichotomized at 25 cm^3^: values < 25 cm^3^ were classified as “low,” and values ≥ 25 cm^3^ were classified as “high.” This exploratory cutoff was internally supported by a log-rank cutoff scan in the high-V_T+N_ subgroup, which identified 85 copies/mL as the optimal cut point, and by bootstrap resampling showing stable risk direction. Additional validation results for the continuous interaction Cox model and the Cox proportional hazards model are provided in the Supplementary Materials (Table S9 and Figure S3).

Cox interaction analysis and risk heatmap suggested a potential antagonistic relationship between baseline ctHPV DNA levels and tumor burden (V_T+N_), whereby the prognostic effect of ctHPV DNA tended to shift from deleterious to protective with increasing tumor burden (Figure 3A,B). To quantitatively assess the interaction, we constructed a multivariable Cox model with an interaction term between ctHPV DNA and tumor burden. The overall model was statistically significant and showed favorable discrimination (C-index, 0.809; LRT *p* = 0.022; log-rank *p* = 0.024, Table S9). In this model, tumor burden was significantly associated with increased PFS risk (HR = 6.09; 95% CI, 1.13–32.76; *p* = 0.035), whereas baseline ctHPV DNA was not significant. The interaction model demonstrated stable directionality in leave-one-out analysis and favorable bootstrap-validated discrimination (mean C-index, 0.809; 95% CI, 0.694–0.916) (Figure S3A,B).

In contrast to the classical linear correlation in progression-free patients, progression cases exhibited an unexpected non-linear relationship between ctHPV DNA and V_T+N_, indicating a tumor volume–ctHPV DNA discordance. In subgroup analysis, each log2 increase in ctHPV DNA was associated with decreased PFS risk in the high V_T+N_ group (HR, 0.809; 95% CI, 0.687–0.952; *p* = 0.011), but not in the low V_T+N_ group (Figure 3C; Table S9). Based on these results, we identified a high-risk subset with dismal survival, characterized by low ctHPV DNA despite high V_T+N_ (VOL^high^DNA^low^). Using an exploratory cutoff of 85 copies/mL and 25 cm^3^ for ctHPV DNA and V_T+N_ respectively, the VOL^high^DNA^low^ subgroup showed significantly worse PFS than the VOL^high^DNA^high^ subgroup (HR = 7.94; 95% CI, 1.98–31.77; *p* = 0.003), and the overall four-group comparison was significant (log-rank *p* = 1.51 × 10^−4^; LRT *p* = 0.002) (Figure 3D; Table S9). The clinical utility of this two-factor risk stratification was further supported by a nomogram incorporating ctHPV DNA and V_T+N_, with a bootstrap-corrected C-index of 0.697 (Figure 3E).

### 3.5. Molecular Relevance of VOL^high^DNA^low^ Phenotype and Gene-Based Survival Prediction

**Figure 4:**
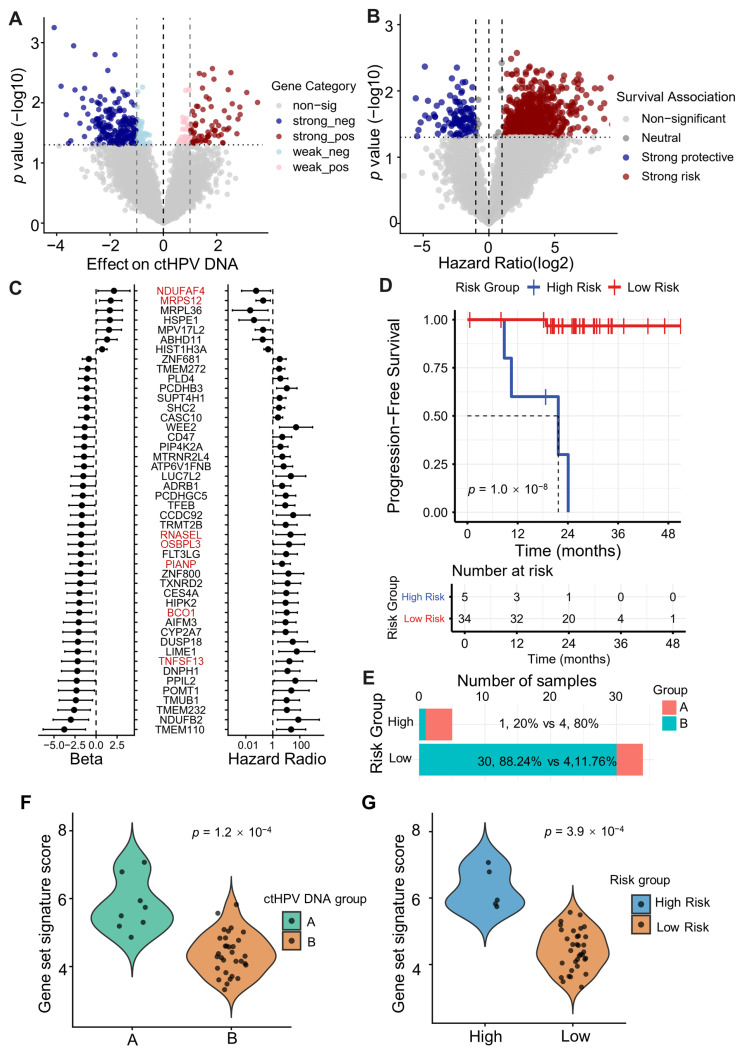
Integrated identification of ctHPV DNA-associated and prognostic gene signature reveals a Ridge–Cox risk model for progression-free survival stratification in high-tumor-burden patients. (**A**) Volcano plot showing genes associated with ctHPV DNA level. The x-axis represents the regression coefficient (β) per log2 TPM. The y-axis shows −log10(*p*-value). The horizontal dotted line indicates *p* = 0.05. Vertical dashed lines indicate β = −1, 0, and 1. (**B**) Volcano plot of gene-level survival associations from univariate Cox regression. The x-axis represents log2(HR). The y-axis represents −log10(*p*-value). The horizontal dotted line indicates *p* = 0.05. Vertical dashed lines correspond to HR = 0.5, 1, and 2. (**C**) Dual Forest plot of genes significant in both ctHPV DNA and PFS models. The left panel shows β (ctHPV DNA model), and the right panel shows HR (PFS model, log scale). Error bars indicate 95% confidence intervals. Dashed vertical lines indicate β = 0 and HR = 1. (**D**) Kaplan–Meier curves of progression-free survival stratified by Ridge–Cox risk score. Log-rank *p*-value is shown. Numbers at risk are displayed below the plot. Genes included in the Ridge–Cox gene signature are highlighted in red text in (**C**). (**E**) Distribution of group A and group B samples within high- and low-risk categories. Bars indicate sample counts, and proportions are annotated. Samples with high tumor burden (V_T+N_ ≥ 25 cm^3^) were divided into two groups: group A, ctHPV DNA < 85 copies/mL, and group B, ctHPV DNA ≥ 85 copies/mL. (**F**) Distribution of gene signature score between group A and group B. Two-sided Wilcoxon rank-sum test *p*-value is shown. (**G**) Distribution of gene signature score between high- and low-risk groups. Two-sided Wilcoxon rank-sum test *p*-value is shown. The C-index of the Ridge–Cox model was 0.939, and the bootstrap-corrected C-index was 0.946. Additional validation results are provided in the Supplementary Materials (Table S9 and Figure S3).

To investigate the molecular basis linking VOL^high^DNA^low^ HPV(+) OPSCC with adverse prognosis, we performed bulk RNA-seq on primary tumor samples, focusing on high V_T+N_ tumors to capture volume–ctHPV DNA discordance. Linear regression identified 300 genes with an expression level strongly correlated with ctHPV DNA load after adjusting for tumor volume (|β| ≥ 1, *p* < 0.05; Figure 4A). Parallel Cox regression yielded 833 genes predictive of PFS (|log_2_(HR)| ≥ 1, *p* < 0.05; Figure 4B) as prognostic indicators. The intersection of both gene sets based on consistent directional effects produced 46 candidates that characterize the molecular features of the DNA^low^-to-prognosis coupling within the high V_T+N_ zone. These candidates predominantly involve processes of transcriptional/epigenetic regulation, mitochondrial metabolism, immune signaling, and membrane trafficking, etc. (Figure 4C).

Furthermore, representative genes were selected by LASSO to construct a Ridge–Cox prognostic model. The resultant seven-gene signature (NDUFAF4, MRPS12, RNASEL, OSBPL3, PIANP, BCO1 and TNFSF13) provided exceptional risk discrimination (C-index, 0.939; AUC for 1-year and 2-year PFS, 0.97 and 0.95 respectively), where high-risk and low-risk subgroups showed significant survival difference (2-year PFS, 30% vs. 96.8%; *p* < 0.001) (Figure 4D). Crucially, the risk categories highly mirrored clinical phenotypes, where 80% of high-risk and 88.2% of low-risk patients fell into the VOL^high^DNA^low^ and VOL^high^DNA^high^ subgroups, respectively (Figure 4E). Intergroup comparison also revealed a significant difference in signature scores between gene-based risk subgroups and clinical phenotypes (Figure 4F,G). These findings demonstrated robustness across internal validation via leave-one-out resampling and calibration plots (Supplementary Figure S3C–E), further supporting VOL^high^DNA^low^ HPV(+) OPSCC as a distinct, molecularly aggressive biological entity.

## 4. Discussion

In the current study, we characterized the different clinical relevance of pretreatment and follow-up ctHPV DNA in HPV(+) OPSCC. Pretreatment ctHPV DNA level strongly correlated with baseline cT, cN, overall stage and tumor volume, particularly with LN-related volumetric and clinicoradiological features on MR and PET/CT, suggesting its role as a surrogate biomarker of pretreatment tumor burden. In comparison, follow-up ctHPV DNA detectability offers significant prognostic value by predicting tumor progression or death. Specifically, we defined a potential subset of HPV(+) OPSCC characterized by high tumor volume but discordantly low ctHPV DNA (VOL^high^DNA^low^). This phenotype showed inferior prognosis in this cohort and was associated with molecular alterations that informed a seven-gene prediction model for PFS, suggesting its potential clinical relevance in HPV(+) OPSCC.

Prior studies have shown that baseline ctHPV DNA levels in HPV(+) OPSCC depend on multiple factors. Earlier studies found that ctHPV DNA load significantly correlated with cTNM stage [20] and total tumor size [21]. In Huttinger et al.’s report, primary (but not nodal) tumor burden was associated with baseline ctHPV DNA score [11]. By contrast, another study detected no such association with cT classification, tumor size or PET parameters [22]. Chera et al. even observed significantly lower baseline ctHPV DNA levels in T3–4 versus T2 tumors [23], highlighting substantial controversy regarding the role of primary tumor in influencing ctHPV DNA load. Recently, growing evidence has pointed to LN-related factors, such as cN, diameter and SUVmax, as predominant determinants of baseline ctHPV DNA [22,24,25,26]. Although SUVmax was included as an exploratory PET-derived parameter, it is sensitive to image noise and does not fully capture whole-tumor metabolic burden. Prior studies have suggested that volumetric PET parameters, such as MTV, may correlate more closely with ctDNA detection or ctHPV DNA level than SUVmax [27]. The current study extends the literature by comprehensively examining the impact of T- and N-related parameters. We found that baseline ctHPV DNA was influenced by three-dimensional V_T_ and V_N_, as well as categorical LN features of anatomical extension. Among these, V_T_ and MLD emerged as independent predictors of ctHPV DNA, suggesting a synergistic contribution from both primary and nodal burden.

Consistent with previous reports [8,24], our study further demonstrated the value of follow-up ctHPV DNA in risk stratification (2-year PFS, 94.0% vs. 68.6%). Although the sensitivity (33.3%) was similar to data from NRG-HN002 (27%) [25] and another prospective pilot study [26], our accuracy was generally lower than in earlier U.S. series, which reported a sensitivity around 90% and a PPV of 79–95% with retrospective [9,11,22] or prospective [8] cohorts. This discrepancy might be attributed to several reasons. First, our ddPCR platform was customized in-house due to the unavailability of commercially available tumor-tissue-modified viral (TTMV)-HPV DNA assays widely adopted in the U.S. Accuracy may be affected by technological nuances in DNA purification efficiency, primer and probe sequences, instrumentation, and HPV genotype coverage (HPV16/18/33, adjusted for East China distribution). In addition, definitive radiotherapy-based treatment may limit posttreatment shedding of ctHPV DNA from irradiated areas, a phenomenon also seen in other cancers [28]. Supporting this, five of six false-negative cases (83.3%) had locoregional relapses in our study. Moreover, single-time-point detectability may be insufficient to detect occult residual or recurrent disease. In nasopharyngeal carcinoma (NPC), two positive EBV DNA tests yielded a higher PPV than single positivity (0.66 vs. 0.10) [29]. Similarly, Chera et al. reported a PPV of 94% with two consecutive positive HPV DNA tests in HPV(+) OPSCC [30], indicating the advantage of serial testing to enhance accuracy. Furthermore, prolonged follow-up may identify more recurrences among the 6 false-positive cases, which might further increase the accuracy of surveillance. The relatively low sensitivity observed in our cohort may also be associated with different induction treatment approaches. Induction chemotherapy and induction chemoimmunotherapy may differentially affect ctHPV DNA kinetics before definitive radiotherapy, thereby influencing posttreatment ctHPV DNA detectability and its performance in relapse prediction. More sensitive HPV-specific sequencing-based assays, repeated serial testing, shorter surveillance intervals, or confirmatory consecutive positive tests may improve the performance of ctHPV DNA surveillance. Future prospective studies should distinguish these induction strategies in patients who complete definitive radiotherapy and incorporate standardized serial ctHPV DNA sampling to determine whether post-induction ctHPV DNA changes can improve subsequent risk prediction.

Follow-up ctDNA performance varies by tumor type, treatment setting, assay platform, sampling schedule, and tumor biology. Highly sensitive assays in non-head-and-neck squamous cell carcinoma (non-HNSCC) settings, including resected colorectal cancer and early breast cancer, have shown that ctDNA can precede radiological or clinical relapse by several months [31,32]. In HNSCC, a tissue-agnostic genome-wide methylome enrichment molecular residual disease (MRD) assay detected recurrence up to 14.9 months before standard examination or imaging, with a mean lead time of 4.1 months [33]. However, lead times in viral and non-viral HNSCC remain heterogeneous. An NPC study with EBV DNA liquid biopsy reported median lead times of 2.5 months for the first detection and 0.8 months for a confirmatory test [29]. A recent systematic review and meta-analysis of non-viral HNSCC reported a median lead time of 4.6 months between ctDNA detection and clinically or radiologically confirmed progression [34]. We observed lead times of ctHPV DNA ranging from 0 to 39 days in this study, comparable to previously reported medians of 47–56 days in HPV(+) OPSCC [8,9,24]. This result should therefore be viewed as an assay- and cohort-specific finding, given the limited number of recurrence events, the use of an in-house ddPCR assay, variable sampling intervals, induction-based treatment, and ctHPV DNA shedding biology. Beyond relapse surveillance, ctDNA may also reflect tumor burden; in HNSCC, ctDNA parameters have been associated with FDG-PET/CT-derived metabolic tumor volume and total lesion glycolysis [35]. Whether HPV-specific sequencing-based ctHPV DNA assays, repeated serial testing, shorter surveillance intervals, confirmatory consecutive positive tests, or integration with imaging biomarkers can improve surveillance performance warrants further investigation [27,36].

Most intriguingly, a potential high-risk VOL^high^DNA^low^ phenotype was identified in our study, reflecting a synergistic effect of ctHPV DNA and tumor volume on prognosis. This adds to prior data and plausibly explains the controversial prognostic value of baseline ctHPV DNA in HPV(+) OPSCC. For instance, while some reports showed no survival impact of ctHPV DNA [10,37], Cao et al. [22] and Adrian et al. [38] revealed a significant association between low pretreatment ctHPV DNA and superior PFS. By contrast, Chera et al. [23] found that lower baseline ctHPV DNA was related to adverse clinical risk factors (T4 disease or >10 pack years of smoking history), although a direct correlation between ctHPV DNA and survival outcomes was not presented. In the current study, we observed that treatment failures clustered predominantly in the subset with high total tumor volume yet paradoxically low ctHPV DNA rather than in those with consistently high ctHPV DNA (VOL^high^DNA^high^) or small volume (VOL^low^), suggesting the importance of the ctHPV DNA–volume combination in prognosis, rather than baseline ctHPV DNA alone. Supporting this, an antagonistic prognostic interaction was detected, wherein low ctHPV DNA was associated with favorable outcomes in low-V_T+N_ patients but paradoxically increased risk in high-V_T+N_ patients. These findings aligned with earlier evidence in cervical cancer that low-level tissue HPV DNA plus larger tumor size defined a high-risk population [39,40], yet contrasted with data from other cancers (i.e., NPC, non-small cell lung cancer) showing that the VOL^high^DNA^high^ subset typically harbors the highest survival risk [15,16], suggesting the biological distinctiveness of HPV-related cancers. Notably, the baseline ctHPV DNA-V_T+N_ combination showed promising risk stratification in this cohort, suggesting that pretreatment integrative biomarkers may provide earlier prognostic information in HPV(+) OPSCC.

The volume–ctHPV DNA discordance reflects the inter-tumor heterogeneity in HPV(+) OPSCC, and the underlying mechanisms warrant further elucidation. One possible explanation is confounding by non-HPV16 genotypes, which have been reported to yield a lower median TTMV-HPV DNA score. However, this is unlikely in our study, as HPV16 was confirmed by PCR-RDB in 97% of patients. Another common explanation is HPV integration, which leads to a reduction in the episomal viral DNA copy number [23] and deterioration of survival [41] via E6/7 overexpression. In the current study, we screened for genes correlated with both ctHPV DNA level and prognosis to explore the molecular underpinnings of the VOL^high^DNA^low^-prognosis association. The identified genes generated several plausible mechanisms: a relatively immune-cold environment (enriched for CD47) that may inhibit DNA digestion and release; enhanced DNA clearance via the autophagy–lysosome pathway (TFEB and TMUB1); and an augmented physical barrier (PCDHB3 and PCDHGC5) that could block DNA shedding into circulation. Although a detailed mechanism exploration is beyond the scope of this study, these findings warrant further investigation in the future.

### Limitations

The in-house ddPCR assay for ctHPV DNA detection is platform-specific and lacks external validation. Although the included HPV genotypes were selected according to the regional distribution of HPV-positive OPSCC, the assay remains limited compared with commercial TTMV-HPV DNA assays or HPV NGS-based platforms. The low number of tumor failures may also limit the power of subgroup prognostic analyses. Moreover, this study could not fully evaluate the effect of induction therapy, especially induction immunotherapy, on ctHPV DNA kinetics. Larger prospective studies with standardized serial ctHPV DNA sampling are needed to validate our findings and to determine whether post-induction ctHPV DNA changes can improve subsequent risk prediction.

## 5. Conclusions

In this HPV-positive OPSCC cohort mainly treated with induction chemotherapy or chemoimmunotherapy followed by definitive radiotherapy, pretreatment ctHPV DNA was closely associated with tumor burden, particularly primary tumor volume and nodal size. Follow-up ctHPV DNA detectability was associated with inferior PFS, although its sensitivity for relapse prediction was limited in this cohort. Integrating baseline ctHPV DNA with total tumor volume identified a potential high-risk VOL^high^DNA^low^ phenotype, suggesting that ctHPV DNA–tumor volume discordance may provide additional prognostic information beyond ctHPV DNA alone. Exploratory RNA-seq analysis was performed to investigate potential biological features underlying this discordance, and a seven-gene signature associated with PFS was identified. These exploratory findings require validation in larger prospective cohorts with standardized imaging assessment and serial ctHPV DNA sampling, especially in patients receiving different induction treatment strategies.

## Figures and Tables

**Table 1:** Baseline Patient characteristics.

Characteristic	Category	Number of Patients (N = 103)	Percentage (%)
Age (years)	≤55	52	50.49
	>55	51	49.51
Sex	Female	19	18.45
	Male	84	81.55
Smoking status	Never	42	40.78
	Former/current	61	59.22
Smoking history (pack-year)	≤10	57	55.34
	>10	46	44.66
Drinking	No	74	71.84
	Yes	29	28.16
T classification	1	30	29.13
	2	36	34.95
	3	21	20.39
	4	16	15.53
N classification (TNM8)	1	52	50.49
	2	38	36.89
	3	13	12.62
N classification (TNM9)	1	18	17.48
	2	53	51.46
	3	32	31.07
Stage (TNM8)	I	37	35.92
	II	40	38.83
	III	25	24.27
	IV	1	0.97
Stage (TNM9)	I	11	10.68
	II	52	50.49
	III	39	37.86
	IV	1	0.97
Radiation therapy ^1^	No or not completed	4	3.88
	Completed	99	96.12
Induction therapy ^1^	No	10	9.71
	Chemotherapy with or without immunotherapy	93	90.29
ctHPV DNA test	Baseline only	15	14.56
	Baseline and Follow-up	88	85.44
Baseline ctHPV DNA detection ^2^	Detectable	83	80.58
	Undetectable	20	19.42
Tumor tissue HPV assessment ^3^	HPV genotype alone	1	0.97
	p16 plus RNA-seq-based HPV detection	4	3.88
	p16 plus HPV genotyping by PCR-RDB	98	95.15
HPV genotype ^4^	HPV16 alone	100	97.09
	Non-HPV16 alone	2	1.94
	HPV16 + HPV18	1	0.97

Abbreviations: PCR-RDB, polymerase chain reaction-reverse dot blot. 1. Patients had radical radiotherapy included in their planned treatment; however, radiotherapy was either not initiated or not completed due to personal reasons. Treatment details and imaging assessment information for the overall cohort are provided in Table S10. 2. All subjects with undetectable ctHPV DNA testing had confirmed detectable HPV DNA in tumor tissue in order to be eligible for cohort inclusion. 3. Tissue HPV genotyping was primarily performed using PCR-reverse dot blot hybridization. For four patients without available PCR-RDB genotyping, HPV16 positivity was confirmed by RNA-seq using tumor tissue samples. 4. In the HPV multi-genotype PCR-RDB assay, two patients (non-HPV16 alone) showed positive results for HPV35 and HPV58. One patient (HPV16 + HPV18) showed double-positive results for HPV16 and HPV18.

## Data Availability

The data supporting the findings of this study are available within the article and its Supplementary Materials. The ctHPV testing reports and RNA-seq data for all samples can be obtained from the corresponding author upon reasonable request, subject to institutional approval and approval by the relevant ethics committee.

## Supplementary Materials

The following supporting information can be downloaded at https://www.mdpi.com/article/10.3390/cancers18142194/s1, Supplementary methods: ctHPV DNA Analysis. Supplementary data: Figure S1: Graphical display of a correlation matrix of all analyzed variables. Spearman’s rank correlation was calculated using pairwise complete observations. Color intensity indicates the strength of correlation (red: positive, blue: negative, white: none). *p*-values were derived from Spearman’s correlation tests for all variable pairs. Figure S2: Violin plots with box plots presenting insignificant difference in ctHPV DNA between patients with cat-egorical variables. Samples were divided into High and Low groups according to baseline ctHPV DNA levels relative to the median. Figure S3: Robustness and Predictive Performance Evaluation of the Cox Models. (A) Leave-one-out sensitivity analysis of the interaction model (ctHPV DNA (log2) × V_T+N_(log2)) in Figure 3A, showing the distribution of hazard ratios for the interaction term upon systematic exclusion of individual cases. (B) Decision curve analysis (DCA) of the interaction model. The net benefit is plotted against a range of threshold probabilities to evaluate clinical utility. (C) Leave-one-out sensitivity analysis of the multi-gene Ridge–Cox signature risk score in Figure 4D. (D) DCA of the Ridge–Cox risk score, comparing the clinical net benefit of the genomic signature with default strategies. (E) Calibration plot of the Ridge–Cox model. The x-axis represents the predicted probability of PFS, and the y-axis represents the observed PFS. The dashed line (45-degree) represents perfect calibration. (F) Time-dependent ROC curves for the Ridge-Cox risk score. The area under the curve (AUC) is provided for the prediction of 1-year and 2-year PFS. Table S1: Baseline Clinical and Imaging Characteristics of the Patients; Table S2: Comparison of mean level of baseline ctHPV DNA among clinical features; Table S3: The difference in the mean value of baseline ctHPV DNA; Table S4: Correlation between baseline ctHPV DNA and analyzed variables; Table S5: Univariable linear regression of baseline ctHPV DNA levels; Table S6: Selection of candidate variables for multivariable regression of baseline ctHPV DNA levels; Table S7: Multivariable linear regression analysis of predictors for baseline ctHPV DNA level; Table S8: Characteristics of individual study subjects with true-positive, false-negative, and false-positive follow-up ctHPV DNA tests; Table S9: Detailed Results of Cox Proportional Hazards Models for PFS; Table S10: Patient imaging assessment and treatment information.

## Abbreviations

The following abbreviations are used in this manuscript:

OPSCC	Oropharyngeal Squamous Cell Carcinoma
ctHPV DNA	Circulating Tumor Human Papillomavirus DNA
PFS	Progression-Free Survival
MLD	Maximum Lymph Node Diameter
iENE	Imaging Extranodal Extension
ddPCR	Droplet Digital PCR
CAT	Categorical Variables
PPV	Positive Predictive Value
NPV	Negative Predictive Value
