# Supplementary material for: Integrating Pretreatment Circulating Tumor HPV DNA and Tumor Volume for Risk Stratification in HPV-Positive Oropharyngeal Squamous Cell Carcinoma
Source: Cancers (Basel). 2026 Jul 8;18(14):2194. doi: 10.3390/cancers18142194 (PMC13407036; doi:10.3390/cancers18142194)
Supplement: Supplementary file 1 [file cancers-18-02194-s001.zip › Supplementary Data_new.pdf]

Table S1. Baseline Clinical and Imaging Characteristics of the Patients.

Variable	Category	Value
**Demographics & History**		
Age (years), n = 103		55.00 ± 8.81
Smoking Amount (pack-year), n = 103		16.24 ± 21.63
**Quantitative Parameters (Continuous)**		
V_T (cm^3), n = 99		15.50 ± 16.14
V_N (cm^3), n = 99		27.16 ± 27.29
V_{T+N} (cm^3), n = 99		42.66 ± 37.28
SUV_{max}-T, n = 64		13.35 ± 5.52
SUV_{max}-N, n = 64		11.17 ± 4.99
MLD (cm), n = 99		3.34 ± 1.95
**Quantitative Parameters (Categorical)**		
SUV_{max}-T, n = 64	>13.35	28 (43.8%)
	≤13.35	36 (56.2%)
SUV_{max}-N, n = 64	>11.17	30 (46.9%)
	≤11.17	34 (53.1%)
V_T (cm^3), n = 99	>15.50	35 (35.4%)
	≤15.50	64 (64.6%)
V_N (cm^3), n = 99	>27.16	37 (37.4%)
	≤27.16	62 (62.6%)
V_{T+N} (cm^3), n = 99	>42.66	37 (37.4%)
	≤42.66	62 (62.6%)
MLD (cm), n = 99	>3.34	53 (53.5%)
	≤3.34	46 (46.5%)
**Nodal Features**		
Number of LNs (continuous), n = 99		4.34 ± 3.51
Number of LNs (categorized), n = 99	>4	40 (40.4%)
	≤4	59 (59.6%)
LN Regions (continuous), n = 99		2.97 ± 1.82
LN Regions (categorized), n = 99	>3	38 (38.4%)
	≤3	61 (61.6%)
LN laterality, n = 99	None	12 (12.1%)
	Unilateral	50 (50.5%)
	Bilateral	37 (37.4%)
Retropharyngeal LN, n = 99	None	65 (65.7%)
	Unilateral	32 (32.3%)
	Bilateral	2 (2.0%)
Necrosis, n = 99	None	39 (39.4%)

	Cystic	12 (12.1%)
	Necrosis	41 (41.4%)
	Cystic and necrosis	7 (7.1%)
iENE, n = 99	G0	37 (37.4%)
	G1	22 (22.2%)
	G2	27 (27.3%)
	G3	13 (13.1%)
**Primary tumor extension**		
Tumor total involved sites, n = 99	>2.00	35 (35.4%)
	≤2.00	64 (64.6%)
Tonsil, n = 99	No	11 (11.1%)
	Yes	88 (88.9%)
Base of tongue, n = 99	No	54 (54.5%)
	Yes	45 (45.5%)
Laryngeal epiglottis, n = 99	No	84 (84.8%)
	Yes	15 (15.2%)
Soft palate, n = 99	No	72 (72.7%)
	Yes	27 (27.3%)
Parapharyngeal space, n = 99	No	73 (73.7%)
	Yes	26 (26.3%)
Medial pterygoid, n = 99	No	90 (90.9%)
	Yes	9 (9.1%)
Extrinsic tongue muscle, n = 99	No	88 (88.9%)
	Yes	11 (11.1%)
Retromolar trigone / pterygomandibular space, n = 99	No	80 (80.8%)
	Yes	19 (19.2%)
Nasopharynx, n = 99	No	91 (91.9%)
	Yes	8 (8.1%)
Bone involvement, n = 99	No	97 (98.0%)
	Yes	2 (2.0%)

*1. Data are presented as mean ± standard deviation (SD) or n (%). 2. Cutoff values were determined by median values. 3.Abbreviations: V_T, volume of primary tumor; V_N, volume of metastatic nodes; V_{T+N} , total of V_T and V_N; MLD, maximum lymph node diameter; SUVmax-T, Maximum standardized uptake value of tumor; SUVmax-N, Maximum standardized uptake value of lymph node; LN, metastatic lymph nodes.

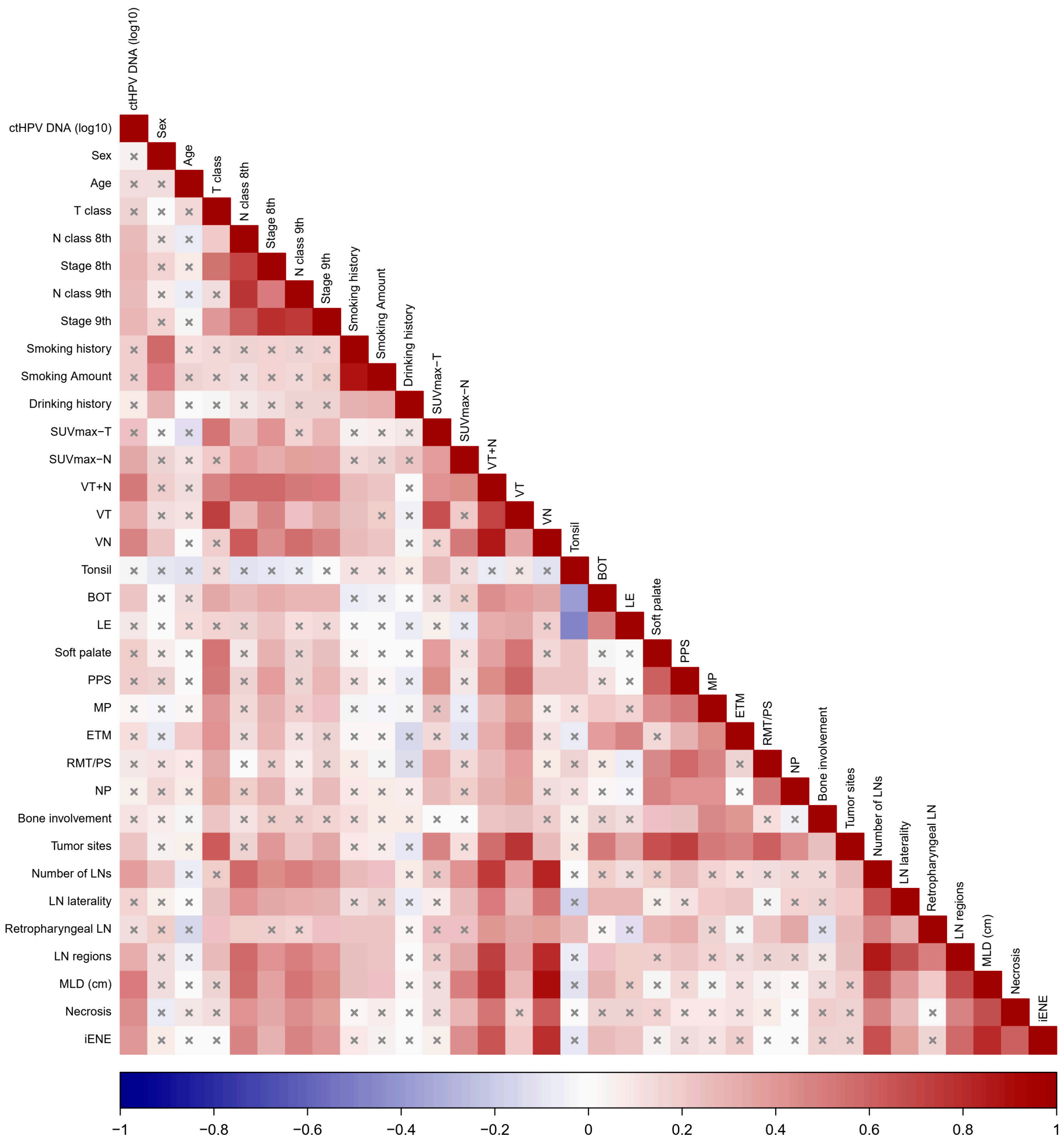

Figure S1. Graphical display of a correlation matrix of all analyzed variables. Spearman's rank correlation was calculated using pairwise complete observations. Color intensity indicates the strength of correlation (red: positive, blue: negative, white: none). P-values were derived from Spearman's correlation tests for all variable pairs.

Table S2. Comparison of mean level of baseline ctHPV DNA among clinical features.

Variable	Category	N	ctHPV DNA (log10) (mean, SD)	Comparison	p_value	q_value	Cohens_d
V_T (cm^3)	>15.50	35	2.696 (1.106)		0.006	0.023	
	≤15.50	64	2.023 (1.193)			0.023	
V_N (cm^3)	>27.16	37	2.877 (0.990)		<0.001	<0.001	
	≤27.16	62	1.893 (1.172)			<0.001	
V_{T+N} (cm^3)	>42.66	37	2.891 (0.991)		<0.001	<0.001	
	≤42.66	62	1.885 (1.165)			<0.001	
SUVmax-N	>11.17	30	2.767 (1.286)		0.023	0.055	
	≤11.17	34	2.084 (1.018)			0.055	
Tumor-involved sites	>2.00	35	2.640 (1.162)		0.020	0.055	
	≤2.00	64	2.053 (1.180)			0.055	
Number of LN	>4.00	40	2.600 (1.215)		0.022	0.055	
	≤4.00	59	2.031 (1.146)			0.055	
MLD (cm)	>3.34	53	2.775 (1.007)		<0.001	<0.001	
	≤3.34	46	1.668 (1.141)			<0.001	
Smoking status	Never	42	1.872 (1.259)		0.036	0.073	
	Former/Current	61	2.400 (1.193)			0.073	
BOT	No	54	2.011 (1.275)		0.020	0.055	
	Yes	45	2.561 (1.044)			0.055	
Stage (TNM8)					0.002	0.011	\
	I	37	1.626 (1.241)				
	II	40	2.474 (1.191)	II vs. I	0.003	0.021	-0.697
	III	25	2.552 (1.084)	III vs. I	0.003	0.021	-0.784
N classification (TNM9)					0.017	0.055	
	I	18	1.884 (1.110)	N1 vs. N3	0.020	0.067	-0.708
	II	53	1.979 (1.255)	N2 vs. N3	0.010	0.041	-0.586
	III	32	2.696 (1.167)				
Stage (TNM9)					0.006	0.023	
	I	11	1.651 (1.188)	I vs. III	0.022	0.067	-0.904
	II	52	1.937 (1.262)	II vs. III	0.004	0.026	-0.609
	III	39	2.667 (1.107)				
LN laterality					<0.001	<0.001	
	None/L0	12	0.937 (0.864)				
	Unilateral/L1	50	2.573 (1.145)	L1 vs. L0	<0.001	<0.001	-1.489
	Bilateral/L2	37	2.269 (1.094)	L2 vs. L0	<0.001	0.002	-1.275
Necrosis					<0.001	0.001	
	None/C0	39	1.645 (1.072)				
	Cystic/C1	12	2.384 (1.028)	C1 vs. C0	0.044	0.112	-0.695

	Necrosis/C2	41	2.662 (1.153)	C2 vs. C0	<0.001	0.001	-0.912
	Cystic and necrosis/C3	7	3.127 (1.015)	C3 vs. C0	0.007	0.035	-1.392
iENE					<0.001	0.003	
	G0	37	1.718 (1.030)				
	G1	22	2.161 (1.372)	G1 vs. G2	0.045	0.112	-0.612
	G2	27	2.898 (1.046)	G0 vs. G2	<0.001	<0.001	-1.138
	G3	13	2.651 (0.968)	G0 vs. G3	0.008	0.035	-0.919

Abbreviations: V_T, volume of primary tumor; V_N, volume of metastatic nodes; V_{T+N}, total of V_T and V_N; SUV_N, maximum standardized uptake value (SUV) of lymph node; LN, metastatic lymph nodes; BOT, base of tongue. MLD, maximal lymph node diameter; iENE, imaging extranodal extension.

Table S3. The difference in the mean value of baseline ctHPV DNA.

Variable	Category	N	ctHPV_DNA_log10_mean_SD	Comparison	p_value	q_value	Cohens_d
Sex	Females	19	2.011 (1.272)		0.513	0.564	
	Males	84	2.224 (1.239)				
Age	>55.00	51	2.254 (1.194)		0.580	0.598	
	≤55.00	52	2.117 (1.296)				
Smoking amount	>16.24	37	2.403 (1.164)		0.172	0.258	
	≤16.24	66	2.062 (1.275)				
Drinking history	Never	74	2.115 (1.211)		0.388	0.474	
	Former/Current	29	2.362 (1.322)				
SUVmax-T	>13.35	28	2.627 (1.299)		0.201	0.276	
	≤13.35	36	2.231 (1.090)				
Number of LN regions	>3.00	38	2.503 (1.234)		0.119	0.219	
	≤3.00	61	2.110 (1.166)			0.219	
T classification					0.134	0.221	
				1 vs. 2	0.994	0.994	0.002
	1	30	1.970 (1.173)	1 vs. 3	0.054	0.130	-0.540
	2	36	1.968 (1.419)	1 vs. 4	0.099	0.205	-0.527
	3	21	2.555 (0.939)	2 vs. 3	0.066	0.158	-0.464
	4	16	2.589 (1.176)	2 vs. 4	0.109	0.217	-0.460
				3 vs. 4	0.925	0.925	-0.032
N classification (TNM8)					0.031	0.067	
	1	52	1.868 (1.195)	1 vs. 2	0.017	0.067	-0.522
	2	38	2.492 (1.199)	1 vs. 3	0.104	0.215	-0.564
	3	13	2.555 (1.314)	2 vs. 3	0.881	0.911	-0.051
Retropharyngeal LN					0.419	0.494	
	None	65	2.158 (1.125)	None vs. Unilateral	0.344	0.448	-0.221
	Unilateral	32	2.425 (1.367)	None vs. Bilateral	0.086	0.185	-0.723
	Bilateral	2	2.966 (0.303)	Unilateral vs. Bilateral	0.156	0.253	-0.402

Abbreviations: SUV_{max}-T, maximum standardized uptake value of tumor; SUV_{max}-N, maximum standardized uptake value of lymph node; CAT, categorized using the median value

as cutoff; V_T, volume of primary tumor; V_N, volume of metastatic nodes; V_{T+N}, total of V_T and V_N; LN, metastatic lymph nodes; MLD, maximum lymph node diameter; BOT, base of tongue; LE, laryngeal epiglottis; SP, soft palate; PPS, parapharyngeal space; MP, medial pterygoid; ETM, extrinsic tongue muscle; RMT_PS, retromolar trigone/pterygomandibular space; NP, nasopharynx.

Table S4. Correlation between baseline ctHPV DNA and analyzed variables.

Variable	Kruskal_H	rho/r	95% CI	p_value	FDR_BH	Bonferroni	Method
Age		0.130	[-0.065, 0.315]	0.189	0.254	1.000	Spearman's rank correlation (bootstrapped CI)
iENE		0.397	[0.234, 0.537]	4.73e-05	2.60e-04	0.002	Spearman's rank correlation (bootstrapped CI)
LN laterality		0.164	[-0.058, 0.350]	0.104	0.158	1.000	Spearman's rank correlation (bootstrapped CI)
MLD (cm)		0.504	[0.338, 0.646]	1.06e-07	2.33e-06	4.66e-06	Spearman's rank correlation (bootstrapped CI)
N classification (TNM8)		0.250	[0.058, 0.419]	0.011	0.030	0.481	Spearman's rank correlation (bootstrapped CI)
N classification (TNM9)		0.263	[0.081, 0.430]	0.007	0.021	0.319	Spearman's rank correlation (bootstrapped CI)
Necrosis		0.440	[0.257, 0.593]	5.21e-06	4.59e-05	2.29e-04	Spearman's rank correlation (bootstrapped CI)
Number of LN regions		0.327	[0.111, 0.529]	9.59e-04	0.004	0.042	Spearman's rank correlation (bootstrapped CI)
Number of LNs		0.378	[0.173, 0.557]	1.14e-04	5.56e-04	0.005	Spearman's rank correlation (bootstrapped CI)
Retropharyngeal LN		0.126	[-0.075, 0.324]	0.213	0.274	1.000	Spearman's rank correlation (bootstrapped CI)
Smoking_Amount		0.184	[-0.011, 0.367]	0.063	0.111	1.000	Spearman's rank correlation (bootstrapped CI)
Stage (TNM8)		0.283	[0.095, 0.456]	0.004	0.013	0.167	Spearman's rank correlation (bootstrapped CI)
Stage (TNM9)		0.293	[0.117, 0.459]	0.003	0.010	0.119	Spearman's rank correlation (bootstrapped CI)
T classification		0.181	[0.001, 0.350]	0.068	0.116	1.000	Spearman's rank correlation (bootstrapped CI)
Tumor-involved sites		0.226	[0.034, 0.415]	0.024	0.051	1.000	Spearman's rank correlation (bootstrapped CI)
V_N (cm^3)		0.487	[0.318, 0.624]	3.20e-07	4.69e-06	1.41e-05	Spearman's rank correlation (bootstrapped CI)
V_T (cm^3)		0.315	[0.137, 0.470]	0.001	0.006	0.065	Spearman's rank correlation (bootstrapped CI)
V_{T+N} (cm^3)		0.512	[0.351, 0.646]	6.28e-08	2.33e-06	2.76e-06	Spearman's rank correlation (bootstrapped CI)
Age_CAT		0.055	[-0.137, 0.238]	0.581	0.608	1.000	Point Biserial Correlation
Bone involvement		0.090	[0.025, 0.173]	0.375	0.424	1.000	Point Biserial Correlation

BOT involvement	0.229	[0.037, 0.390]	0.022	0.049	0.990	Point Biserial Correlation
Drinking history	0.090	[-0.124, 0.289]	0.367	0.424	1.000	Point Biserial Correlation
ETM involvement	0.123	[-0.019, 0.265]	0.224	0.274	1.000	Point Biserial Correlation
LE involvement	0.123	[-0.039, 0.276]	0.224	0.274	1.000	Point Biserial Correlation
MLD_CAT	0.462	[0.305, 0.598]	1.50e-06	1.65e-05	6.59e-05	Point Biserial Correlation
MP involvement	0.018	[-0.177, 0.192]	0.862	0.862	1.000	Point Biserial Correlation
NP involvement	0.073	[-0.136, 0.260]	0.470	0.517	1.000	Point Biserial Correlation
Number of LN regions_CAT	0.160	[-0.065, 0.346]	0.114	0.167	1.000	Point Biserial Correlation
Number of LNs_CAT	0.234	[0.028, 0.399]	0.020	0.049	0.873	Point Biserial Correlation
PPS involvement	0.184	[-0.028, 0.373]	0.068	0.116	1.000	Point Biserial Correlation
RMT_PS involvement	0.102	[-0.094, 0.304]	0.313	0.372	1.000	Point Biserial Correlation
Sex	0.067	[-0.120, 0.254]	0.502	0.538	1.000	Point Biserial Correlation
Smoking history	0.210	[0.032, 0.380]	0.033	0.067	1.000	Point Biserial Correlation
Smoking_Amount_CAT	0.132	[-0.058, 0.308]	0.182	0.254	1.000	Point Biserial Correlation
SP involvement	0.167	[-0.067, 0.393]	0.098	0.158	1.000	Point Biserial Correlation
SUV_{max}-N_CAT	0.288	[0.035, 0.501]	0.021	0.049	0.925	Point Biserial Correlation
SUV_{max}-T_CAT	0.166	[-0.102, 0.405]	0.191	0.254	1.000	Point Biserial Correlation
Tonsil involvement	-0.045	[-0.186, 0.089]	0.655	0.670	1.000	Point Biserial Correlation
Tumor-involved sites_CAT	0.235	[0.048, 0.412]	0.019	0.049	0.851	Point Biserial Correlation
V_N_CAT	0.398	[0.238, 0.538]	4.50e-05	2.60e-04	0.002	Point Biserial Correlation
V_T_CAT	0.269	[0.090, 0.431]	0.007	0.021	0.308	Point Biserial Correlation
V_{T+N}_CAT	0.407	[0.244, 0.549]	2.91e-05	2.14e-04	0.001	Point Biserial Correlation

SUV_{max}-N		0.259	[0.019, 0.492]	0.039	0.074	1.000	Pearson correlation
SUV_{max}-T		0.205	[-0.070, 0.447]	0.104	0.158	1.000	Pearson correlation
Age_CAT	0.338			0.561	0.658818	1	Kruskal-Wallis H test
Bone_CAT	1.092			0.296	0.420594	1	Kruskal-Wallis H test
BOT_CAT	4.705			0.030	0.081218	0.81218338	Kruskal-Wallis H test
ETM_CAT	1.317			0.251	0.376829	1	Kruskal-Wallis H test
iENE_CAT	14.514			0.000	0.000751	0.00375575	Kruskal-Wallis H test
LE_CAT	0.973			0.324	0.436177	1	Kruskal-Wallis H test
LN_laterality_CAT	0.006			0.937	0.936556	1	Kruskal-Wallis H test
MLD_CAT	20.562			0.000	0.000156	0.00015591	Kruskal-Wallis H test
MP_CAT	0.082			0.775	0.804539	1	Kruskal-Wallis H test
Necrosis_CAT	14.952			0.000	0.000745	0.00297802	Kruskal-Wallis H test
NP_CAT	0.349			0.555	0.658818	1	Kruskal-Wallis H test
Number of LN regions_CAT	2.911			0.088	0.169695	1	Kruskal-Wallis H test
Number_LNs_CAT	5.988			0.014	0.048605	0.38883717	Kruskal-Wallis H test
PPS_CAT	3.406			0.065	0.134878	1	Kruskal-Wallis H test
Retropharyngeal_LN_CAT	1.418			0.234	0.371307	1	Kruskal-Wallis H test
RMT_PS_CAT	0.913			0.339	0.436177	1	Kruskal-Wallis H test
Sex_CAT	0.211			0.646	0.726524	1	Kruskal-Wallis H test
Smoking_Amount_CAT	1.745			0.186	0.314663	1	Kruskal-Wallis H test
Smoking_history_CAT	3.614			0.057	0.130874	1	Kruskal-Wallis H test
SP_CAT	3.589			0.058	0.130874	1	Kruskal-Wallis H test

SUV_{max}-N_CAT	6.400	0.011	0.044017	0.308119	Kruskal-Wallis H test
SUV_{max}-T_CAT	2.217	0.136	0.245675	1	Kruskal-Wallis H test
Tonsil_CAT	0.119	0.730	0.788233	1	Kruskal-Wallis H test
Tumor_sites_CAT	4.989	0.026	0.076546	0.68891172	Kruskal-Wallis H test
V_N_CAT	15.783	0.000	0.00064	0.00191853	Kruskal-Wallis H test
V_T_CAT	6.476	0.011	0.044017	0.29527115	Kruskal-Wallis H test
V_{T+N}_CAT	16.657	0.000	0.000605	0.00120937	Kruskal-Wallis H test

Abbreviations: MLD, maximum lymph node diameter; CAT, categorized using the median value as cutoff; V_T, volume of primary tumor; V_N, volume of metastatic nodes; V_{T+N}, total of V_T and V_N; SUV_{max}-T, maximum standardized uptake value of tumor; SUV_{max}-N, maximum standardized uptake value of lymph node; LN, metastatic lymph nodes; BOT, base of tongue; LE, laryngeal epiglottis; SP, soft palate; PPS, parapharyngeal space; MP, medial pterygoid; ETM, extrinsic tongue muscle; RMT_PS, retromolar trigone/pterygomandibular space; NP, nasopharynx; iENE, imaging extranodal extension.

Table S5. Univariable linear regression of baseline ctHPV DNA levels.

Variable	Estimate	95% CI	AIC	p_value	q-value	Shapiro-wilk test for normality of residuals (P value)
Age	0.021	-0.006 - 0.049	339.599	0.132	0.194	0.059
Bone involvement	0.765	-0.940 - 2.470	321.493	0.375	0.435	0.017
BOT involvement	0.550	0.079 - 1.021	316.957	0.022	0.049	0.055
Drinking history	0.247	-0.293 - 0.787	341.087	0.367	0.435	0.008
ETM involvement	0.469	-0.291 - 1.230	320.780	0.224	0.281	0.018
iENE	0.418	0.209 - 0.627	307.354	<0.001	<0.001	0.012
LE involvement	0.412	-0.255 - 1.078	320.779	0.224	0.281	0.012
LN laterality	0.379	0.020 - 0.738	317.909	0.039	0.068	0.109
MLD (cm)	0.317	0.210 - 0.423	291.840	<0.001	<0.001	0.011
MLD_CAT	1.107	0.679 - 1.535	298.550	<0.001	<0.001	0.006
MP involvement	0.074	-0.764 - 0.912	322.268	0.862	0.862	0.017
N classification_TNM8	0.424	0.085 - 0.763	335.833	0.015	0.041	0.003
N classification_TNM9	0.452	0.107 - 0.798	335.263	0.011	0.032	0.006
Necrosis	0.499	0.288 - 0.710	301.996	<0.001	<0.001	0.020
NP involvement	0.322	-0.560 - 1.203	321.764	0.470	0.517	0.012
Number of LN regions	0.217	0.091 - 0.343	310.964	<0.001	0.004	0.021
Number of LN regions_CAT	0.393	-0.096 - 0.882	319.732	0.114	0.172	0.015
Number of LNs	0.121	0.057 - 0.186	309.003	<0.001	0.002	0.023
Number of LNs_CAT	0.570	0.092 - 1.047	316.733	0.020	0.049	0.016
PPS involvement	0.500	-0.038 - 1.038	318.892	0.068	0.116	0.025
Retropharyngeal LN	0.300	-0.158 - 0.758	320.589	0.197	0.262	0.015
RMT_PS involvement	0.311	-0.298 - 0.919	321.254	0.313	0.383	0.024
Sex	0.213	-0.414 - 0.841	341.458	0.502	0.538	0.006
Smoking amount	0.005	-0.007 - 0.016	341.246	0.417	0.471	0.018
Smoking histroy	0.528	0.043 - 1.013	337.280	0.033	0.064	0.014
SP involvement	0.449	-0.084 - 0.983	319.484	0.098	0.159	0.011
Stage_TNM8	0.457	0.163 - 0.750	332.630	0.003	0.010	0.008

Stage_TNM9	0.542	0.190 - 0.894	332.811	0.003	0.010	0.013
SUV_{max}-N	0.062	0.003 - 0.121	204.708	0.039	0.068	0.003
SUV_{max}-N_CAT	0.683	0.106 - 1.259	203.613	0.021	0.049	0.001
SUV_{max}-T	0.044	-0.009 - 0.098	206.404	0.104	0.164	0.073
SUV_{max}-T_CAT	0.395	-0.202 - 0.992	207.370	0.191	0.262	0.065
T classification	0.247	0.016 - 0.478	337.429	0.036	0.068	0.003
Tonsil involvement	-0.173	-0.939 - 0.593	322.094	0.655	0.670	0.014
Tumor-involved sites	0.139	0.015 - 0.263	317.386	0.029	0.060	0.024
Tumor-involved sites_CAT	0.587	0.097 - 1.077	316.687	0.019	0.049	0.036
V_N (cm^3)	0.017	0.009 - 0.025	306.119	<0.001	<0.001	0.022
V_N_CAT	0.984	0.527 - 1.440	305.220	<0.001	<0.001	0.010
V_T (cm^3)	0.023	0.009 - 0.038	311.906	0.001	0.006	0.025
V_T_CAT	0.674	0.188 - 1.159	314.838	0.007	0.022	0.020
V_{T+N} (cm^3)	0.014	0.008 - 0.019	302.989	<0.001	<0.001	0.023
V_{T+N}_CAT	1.006	0.551 - 1.460	304.373	<0.001	<0.001	0.012

Abbreviations: LN, metastatic lymph nodes; CAT, categorized using the median value as cutoff; MLD, maximum lymph node diameter; V_T, volume of primary tumor; V_N, volume of metastatic nodes; V_{T+N}, total of VT and VN; iENE, imaging extranodal extension; SUV_{max}-T, maximum standardized uptake value of tumor; SUV_{max}-N, maximum standardized uptake value of lymph node; BOT, base of tongue; LE, laryngeal epiglottis; SP, soft palate; PPS, parapharyngeal space; MP, medial pterygoid; ETM, extrinsic tongue muscle; RMT_PS, retromolar trigone/pterygomandibular space; NP, nasopharynx.

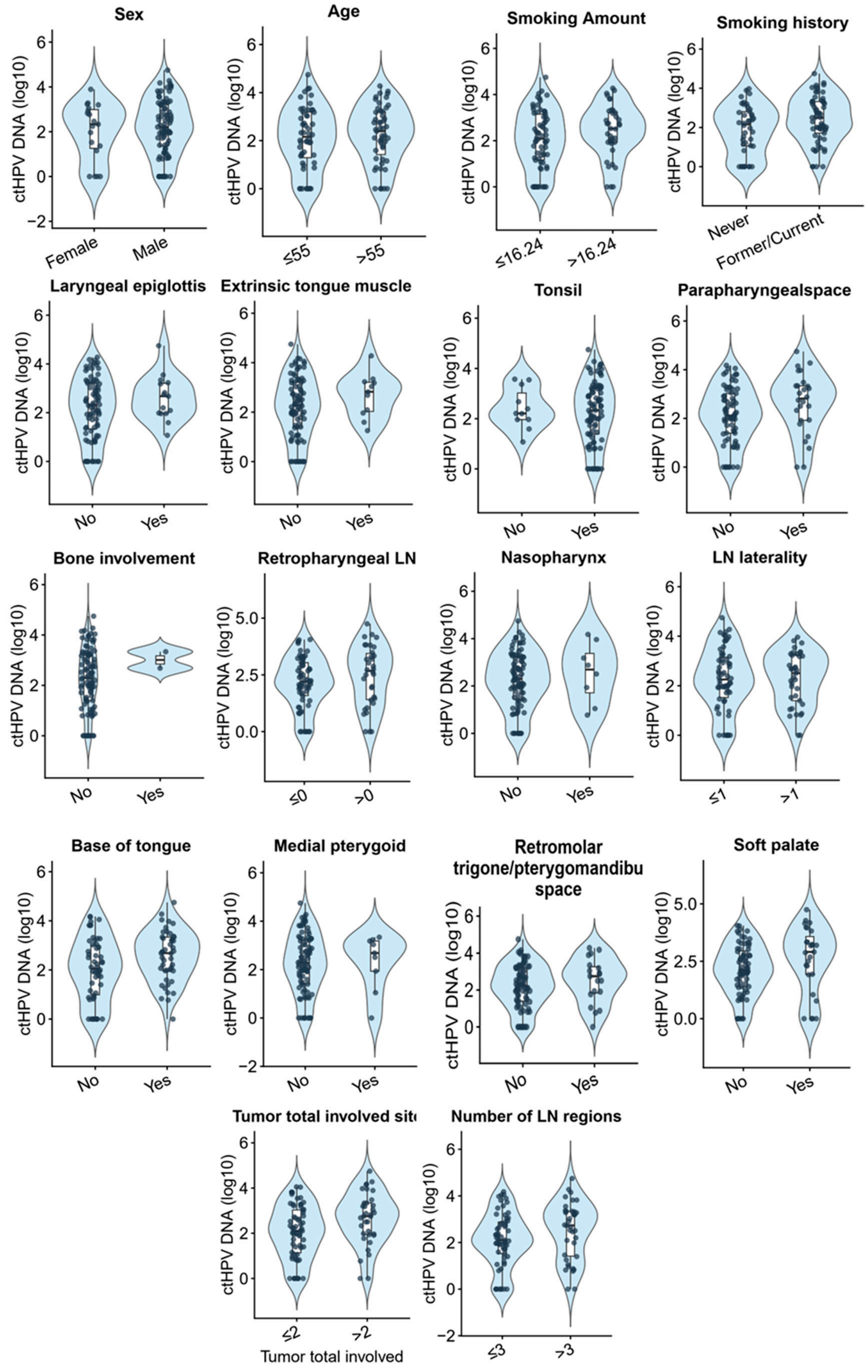

Figure S2. Violin plots with box plots presenting insignificant difference in ctHPV DNA

between patients with categorical variables. Samples were divided into High and Low groups according to baseline ctHPV DNA levels relative to the median. LN: metastatic lymph nodes.

Table S6. Selection of candidate variables for multivariable regression of baseline ctHPV DNA levels.

Variable	**Significant result = 1 Insignificant result = 0**			Score
	Correlation	Difference	Univariable_regression	
MLD_CAT	1	1	1	3
Necrosis	1	1	1	3
Stage_TNM8	1	1	1	3
Stage_TNM9	1	1	1	3
V_N_CAT	1	1	1	3
V_T_CAT	1	1	1	3
V_{T+N}_CAT	1	1	1	3
BOT involvement	1	0	1	2
iENE	1	0	1	2
MLD (cm)	1	0	1	2
N classification_TNM8	1	0	1	2
N classification_TNM9	1	0	1	2
Number of LN Regions	1	0	1	2
Number of LNs	1	0	1	2
Number of LNs_CAT	1	0	1	2
SUV_{max}-N_CAT	1	0	1	2
Tumor-involved sites_CAT	1	0	1	2
V_N (cm^3)	1	0	1	2
V_T (cm^3)	1	0	1	2
V_{T+N} (cm^3)	1	0	1	2
LN laterality	0	1	0	1
Age	0	0	0	0
Bone involvement	0	0	0	0
Drinking history	0	0	0	0
ETM involvement	0	0	0	0
LE involvement	0	0	0	0
MP involvement	0	0	0	0
NP involvement	0	0	0	0
Number of LN Regions_CAT	0	0	0	0
PPS involvement	0	0	0	0
Retropharyngeal LN	0	0	0	0
RMT_PS involvement	0	0	0	0
Sex	0	0	0	0
Smoking amount	0	0	0	0
Smoking history	0	0	0	0
SP involvement	0	0	0	0
SUV_{max}-N	0	0	0	0
SUV_{max}-T	0	0	0	0
SUV_{max}-T_CAT	0	0	0	0

T classification	0	0	0	0
Tonsil involvement	0	0	0	0
Tumor-involved sites	0	0	0	0

Abbreviations: LN, metastatic lymph nodes; CAT, categorized using the median value as cutoff; MLD, maximum lymph node diameter; V_T, volume of primary tumor; V_N, volume of metastatic nodes; V_{T+N}, total of VT and VN; iENE, imaging extranodal extension; SUV_{max}-T, maximum standardized uptake value of tumor; SUV_{max}-N, maximum standardized uptake value of lymph node; BOT, base of tongue; LE, laryngeal epiglottis; SP, soft palate; PPS, parapharyngeal space; MP, medial pterygoid; ETM, extrinsic tongue muscle; RMT_PS, retromolar trigone/pterygomandibular space; NP, nasopharynx.

Table S7. Multivariable linear regression analysis of predictors for baseline ctHPV DNA level.

Variable	Estimate	95% CI	p value	q_value	T value
(Intercept)	1.113	0.697 - 1.529	7.09e-07	2.13e-06	5.309
MLD	0.284	0.173 - 0.394	1.71e-06	2.56e-06	5.100
V_T	0.013	-0.001 - 0.026	0.060	0.060	1.905

Abbreviations: V_T, volume of primary tumor; MLD, maximal lymph node diameter.

Table S8. Characteristics of individual study subjects with true-positive, false-negative, and false-positive follow-up ctHPV DNA tests.

ID	Stage (AJCC 9th)	Baseline ctHPV DNA	HPV genotype	Days from first detectable post-treatment ctHPV DNA to progression or last follow-up	First detectable post-treatment ctHPV DNA(Copies/ml)	Patterns of failure	Clinical status at last follow-up
Group B: True positive (N = 3, 33% of 9 subjects with recurrence)							
B1	T4N3	480.95	HPV16	39	949.00	Distant	Died of disease
B2	T3N3	289.41	HPV16	0	16.06	Local and Regional	Died of disease
B3	T4N2	16.87	HPV16	29	17.27	Regional and distant	Died of disease
Group C: False negative (N = 6, 67% of 9 suCjects with recurrence)							
C1	T2N2	Undetectable	HPV16	n/a	n/a	Regional	NED
C2	T3N1	37.95	HPV16	n/a	n/a	Local	Alive with disease
C3	T2N1	84.51	HPV16	n/a	n/a	Regional	NED
C4	T1N3	Undetectable	HPV16	n/a	n/a	Local	Alive with disease
C5	T4N3	319.48	HPV16	n/a	n/a	Distant	Alive with disease
C6	T3N2	Undetectable	HPV16	n/a	n/a	Local	Alive with disease
Group D: False positive (N = 6, 7.9% of 76 subjeDts without reDurrenDe)							
D1	T3N3	122.17	HPV16	882	49.80	n/a	NED at 51 months
D2	T4N3	1691.3	HPV16	742	128.20	n/a	NED at 38 months
D3	T2N3	28.05	HPV16	857	11.40	n/a	NED at 41 months
D4	T2N2	Undetectable	HPV16	903	10.92	n/a	NED at 37 months
D5	T2N1	Undetectable	HPV16	787	46.63	n/a	NED at 33 months
D6	T2N3	7816.16	HPV16	479	10.74	n/a	NED at 19 months

Abbreviations: NED, no evidence of disease; n/a, not applicable
HPV type from plasma and/or tumor tissue.

Table S9. Detailed Results of Cox Proportional Hazards Models for PFS.

Model & Variables	Figures	β (Coef)	Hazard Ratio (95% CI)	Wald p	Log-rank p	LRT p
**Model 1: Binary Status**	Figure 2A					
Detectable vs. Undetectable ctHPV DNA		1.646	5.186 (1.238–21.725)	0.024	0.012	0.043
**Model 2: Interaction Model**	Figure 3A				0.024	0.022
ctHPV DNA		0.808	2.243 (0.634–7.938)	0.21	/	/
V_{T+N}		1.807	6.093 (1.133–32.763)	0.035	/	/
Interaction (ctHPV DNA × V_{T+N})		-0.192	0.826 (0.649–1.050)	0.118	/	/
**Model 3: Subgroup Analysis**	Figure 3C					
ctHPV DNA (group VOL^{high})		-0.212	0.809 (0.687–0.952)	0.011	/	/
ctHPV DNA (group VOL^{low})		0.571	1.770 (0.733–4.260)	0.205	/	/
**Model 4: Combined Groups**	Figure 3D					
Overall				0.020	1.51E-04	0.002
$VOL^{high}DNA^{low}$ vs. $VOL^{high}DNA^{high}$		2.071	7.936 (1.982–31.774)	0.003	/	/
$VOL^{low}DNA^{high}$ vs. $VOL^{high}DNA^{high}$		0.182	1.199 (0.125–11.535)	0.875	/	/
$VOL^{low}DNA^{low}$ vs. $VOL^{high}DNA^{high}$		/	Not estimable due to sparse events	/	/	/
**Model 5: Continuous Variables**	Figure 3E					

ctHPV DNA		-0.627	0.534 (0.266–1.074)	0.078		0.157
V_{T+N}		0.462	1.587 (0.919–2.742)	0.097		0.157
**Model 6: Risk Stratification**	Figure 4D					
Risk Group (Low vs. High)		-3.861	0.021 (0.002–0.194)	7.00E-04	1.00E-08	1.00E-04

Abbreviations: β, regression coefficient; HR, hazard ratio; CI, confidence interval; LRT, Likelihood Ratio Test.

Likelihood Ratio Test (LRT) p: The p-value refers to the global significance of the entire model.

Model 4 Stability: The Hazard Ratio and its 95% CI are denoted as “>100" due to "Complete Separation" (lack of events in the reference group or specific strata). In such cases, the Wald test p (0.999) is statistically unreliable; the significance should be interpreted via the Log-rank or LRT p-values.

Variables: Log-transformation was applied to quantitative variables (ctHPV DNA and V_{T+N}) to satisfy model assumptions.

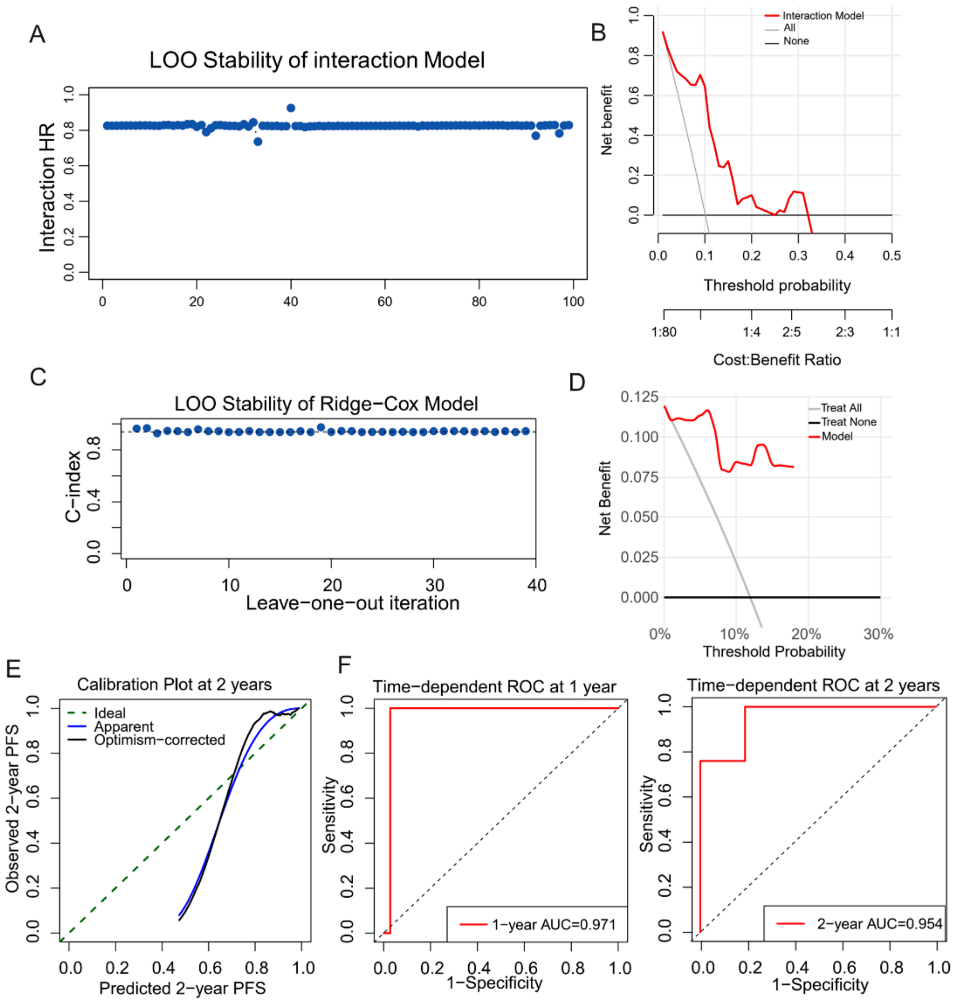

Figure S3. Robustness and Predictive Performance Evaluation of the Cox Models. A. Leave-one-out sensitivity analysis of the interaction model (ctHPV DNA (log2) × V_{T+N}(log2)) in Figure 3A, showing the distribution of hazard ratios for the interaction term upon systematic exclusion of individual cases. B. Decision curve analysis (DCA) of the interaction model. The net benefit is plotted against a range of threshold probabilities to evaluate clinical utility. C. Leave-one-out sensitivity analysis of the multi-gene Ridge−Cox signature risk score in Figure 4D.D. DCA of the Ridge−Cox risk score, comparing the clinical net benefit of the genomic signature with default strategies. E. Calibration plot of the Ridge−Cox model. The x-axis represents the predicted probability of progression-free survival (PFS), and the y-axis represents the observed PFS. The dashed line (45-degree) represents perfect calibration. F. Time-dependent ROC curves for the Ridge-Cox risk score. The area under the curve (AUC) is provided for the prediction of 1-year and 2-year PFS.

Table S10. Patient imaging assessment and treatment information.

Characteristic	Category	Number of patients (N = 103)	Percentage (%)
Induction chemotherapy (IC)	None / not recorded	10	9.7
	TP (docetaxel + cisplatin)	88	85.4
	TC (docetaxel + carboplatin)	3	2.9
	PF (cisplatin + fluorouracil)	2	1.9
IC cycles	0	10	9.7
	1	3	2.9
	2	87	84.5
	3	3	2.9
Induction immunotherapy	None	20	19.4
	Toripalimab	81	78.6
	Tislelizumab	2	1.9
Immunotherapy cycles	0	20	19.4
	1	2	1.9
	2	81	78.6
RT regimen[1]	No	2	1.9
	Not completed	2	1.9
	60/30	48	46.6
	66/30	3	2.9
	66/33	3	2.9
	70/35	45	43.7
Concurrent chemotherapy	No	55	53.4
	Yes	48	46.6
MRI[2]	No	1	1.0
	Yes	102	99.0
CT[3]	No	11	10.7
	Yes	92	89.3
18F-FDG PET/CT	No	35	34.0
	Yes	68	66.0

1. Patients who did not complete radiotherapy had no documented records of receiving any subsequent antitumor treatment during follow-up.
2. Accessible pretreatment baseline MRI scan of oropharynx and neck for extraction of radiomics features, including axial contrast-enhanced T1 weighted imaging (CE-T1WI) and T2 weighted imaging (T2WI).
3. CT refers to contrast-enhanced computed tomography.

Bulk sequencing methods

RNA sequencing (RNA-seq)

Sample	Number of input reads	Uniquely mapped reads number	Uniquely mapped reads %	Average mapped length	Mismatch rate per base, %	% of reads mapped to multiple loci	% of reads mapped to too many loci
OPC_080	35571013	32262336	90.70%	298.18	0.18%	2.22%	0.01%
OPC_072	27762722	26344825	94.89%	297.81	0.19%	2.91%	0.02%
OPC_058	36480547	34051647	93.34%	295.63	0.18%	4.16%	0.02%
OPC_014	39761482	37562229	94.47%	288.06	0.48%	3.23%	0.02%
OPC_054	40239537	38316884	95.22%	295.2	0.17%	3.21%	0.02%
OPC_060	41078912	38978825	94.89%	295.42	0.17%	3.52%	0.02%
OPC_033	23380146	22018459	94.18%	292.34	0.28%	2.20%	0.02%
OPC_067	34970439	32945922	94.21%	294.55	0.20%	3.85%	0.02%
OPC_056	34980023	32853666	93.92%	295.46	0.18%	3.92%	0.02%
OPC_055	35371449	33511104	94.74%	294.7	0.16%	3.09%	0.01%
OPC_029	35266688	28967539	82.14%	296.28	0.22%	2.79%	0.02%
OPC_043	39548511	34317378	86.77%	294.65	0.17%	9.74%	0.02%
OPC_066	36604055	34551300	94.39%	293.77	0.18%	3.97%	0.02%
OPC_087	30788009	29357442	95.35%	297.39	0.20%	2.88%	0.02%
OPC_063	39672917	37724956	95.09%	295.91	0.16%	3.42%	0.02%
OPC_075	35498352	34070224	95.98%	298.34	0.18%	2.68%	0.02%
OPC_064	42771162	39738953	92.91%	294.68	0.19%	4.65%	0.02%
OPC_083	37707959	36093381	95.72%	297.23	0.18%	2.93%	0.02%
OPC_026	27314751	25068541	91.78%	260	0.58%	2.90%	0.03%
OPC_069	35035878	33680664	96.13%	297.77	0.18%	2.52%	0.02%
OPC_074	34274211	32280216	94.18%	297.66	0.19%	3.22%	0.01%
OPC_084	30413791	28615167	94.09%	297.99	0.18%	2.61%	0.02%
OPC_016	30888357	27825055	90.08%	265.31	0.53%	3.04%	0.03%
OPC_076	34520107	33186542	96.14%	298.51	0.17%	2.48%	0.02%
OPC_032	34496356	32433007	94.02%	296.43	0.24%	2.53%	0.02%
OPC_068	32476493	30726625	94.61%	297.64	0.20%	2.87%	0.03%
OPC_082	41728767	40183751	96.30%	298.37	0.17%	2.35%	0.02%
OPC_085	35039108	33350877	95.18%	297.62	0.18%	2.83%	0.01%
OPC_078	37636916	36115173	95.96%	298.29	0.17%	2.68%	0.02%
OPC_053	50370962	29498282	58.56%	296.12	0.18%	2.05%	0.01%
OPC_035	17982481	16995550	94.51%	293.65	0.31%	2.09%	0.01%
OPC_042	38956938	34730967	89.15%	293.85	0.18%	8.47%	0.02%
OPC_065	39508721	37223049	94.21%	294.91	0.18%	3.99%	0.02%
OPC_036	27073977	25503686	94.20%	292.43	0.34%	2.39%	0.02%

OPC_070	30528514	21980038	72.00%	297.72	0.17%	2.11%	0.01%
OPC_077	39393206	38003887	96.47%	298.44	0.17%	2.40%	0.02%
OPC_023	40090342	37640020	93.89%	279.87	0.57%	3.02%	0.02%
OPC_031	22678590	21550215	95.02%	295.83	0.26%	2.54%	0.02%
OPC_034	20560438	17334962	84.31%	293.13	0.35%	2.51%	0.01%

*Reference genome was GRCh38.
