# Supplementary material for: Integrating Pretreatment Circulating Tumor HPV DNA and Tumor Volume for Risk Stratification in HPV-Positive Oropharyngeal Squamous Cell Carcinoma
Source: Cancers (Basel). 2026 Jul 8;18(14):2194. doi: 10.3390/cancers18142194 (PMC13407036; doi:10.3390/cancers18142194)
Supplement: Supplementary file 1 [file cancers-18-02194-s001.zip › Supplementary Methods.pdf]

ctHPV DNA Analysis

I. Plasma Separation

1、 Centrifuge the blood sample at 1600g for 10 minutes to remove cells.

2、Transfer the supernatant to a new 1.5 ml centrifuge tube, taking care not to aspirate the precipitate.

2、 Centrifuge at 16000g for 10 minutes to remove cell debris.

4、Aspirate the supernatant and transfer it to a new 1.5 ml centrifuge tube, being careful not to aspirate the precipitate.

5、If it is to be used on the same day, store it briefly at 4°C. Otherwise, store the collected plasma at -80°C.

II. Extraction of Plasma ctHPV DNA

Name: Column-based Cell-free DNA Extraction Kit (Magen)
Item number: IVD3182, Version: ACB2

Plasma DNA Extraction (taking the extraction of 2 ml as an example)

1、 Transfer 200 μl of Proteinase K to a 50 ml centrifuge tube.

2、 Transfer 2 ml of serum, plasma or other liquid sample to the centrifuge tube containing Proteinase K and mix for 5 seconds.

3、 Add 1.6 ml of Buffer ACL/Carrier RNA (1μg) to the sample, vortex for 15 seconds. Incubate in a water bath at 60°C for 30 minutes, and invert it several times occasionally for mixing.

4、 Add 3.6 ml of Buffer ACB2 to the sample. Vortex for 15 seconds. Place on ice for 5 minutes.

5、 Connect the HiPure ctHPV DNA Mini Column and Vac-Connectors to the vacuum filtration unit.

6、 Insert the Extender Tubes into the column.

7、 Transfer the mixture obtained in step 4 to the column and turn on the vacuum pump for filtration. Continue to transfer the mixture to the column for filtration until all the mixture is transferred to the column and the filtration is completed. Turn off the vacuum pump and let the pressure drop to zero.

8、 Remove the column from the filtration unit. Place the column back into the collection tube. Add 900 μl of Buffer DCW1 to the column. Centrifuge at 13,000 x g for 60 seconds.

9、 Discard the filtrate, place the column back into the collection tube. Add 900 μl of Buffer DCW2 to the column. Centrifuge at 13,000 x g for 60 seconds.

10、 Discard the filtrate, place the column back into the collection tube. Add 900 μl of absolute ethanol to the column. Centrifuge at 13,000 x g for 60 seconds.

11、 Discard the filtrate, place the column back into the collection tube. Centrifuge at 13,000 x g for 3 minutes.

12、 Remove the column and place it in a new 1.5 ml collection tube, then place it in an oven at 56°C for 10 minutes to dry.

13、 Add 50-60 μl of Nuclease Free Water or Buffer TE to the center of the membrane of the column. Let it stand for 3 minutes. Centrifuge at ≥13,000 × g for 1 minute.

14、 Transfer the eluate to the center of the membrane of the column. Let it stand for 1 minute. Centrifuge at ≥13,000 × g for 1 minute.

15、 Discard the DNA binding column, measure the DNA concentration by Qubit, and store the DNA at -20°C or -80°C.

III. Primer and Probe Sequences:

HPV16 Forward Primer (HPV16-E6-F1): 5'-TATGCACAGAGCTGCAAACA-3' (SEQ ID No: 1)

HPV16 Reverse Primer (HPV16-E6-R1): 5'-GCAAAGTCATATACCTCACGTC-3' (SEQ ID No: 2)

HPV16 Probe (HPV16-E6-P1 (FAM)): 5'-TGTGTACTGCAAGCAACAGTTACTG-3' (SEQ ID No: 3)

HPV18 Forward Primer (HPV18-F1): 5'-TGAAGCCAGAATTGAGCTAG-3' (SEQ ID No: 4)

HPV18 Reverse Primer (HPV18-R1): 5'-AGGACAGGGTGTTCAGAA-3' (SEQ ID No: 5)

HPV18 Probe (HPV18-P1 (FAM)): 5'-TCAGCAGACGACCTTCGA-3' (SEQ ID No: 6)

HPV33 Forward Primer (HPV33-E6-F1): 5'-CCACAGTTCGTTTATGTGTCA-3' (SEQ ID No: 7)

HPV33 Reverse Primer (HPV33-E6-R1): 5'-TGCCCATAAGTAGTTGCTGT-3' (SEQ ID No: 8)

HPV33 Probe (HPV33-E6-P2-Low (FAM)): 5'-AGTACAGCA(ZEN)AGTGACCTACG-3' (SEQ ID No: 9)

GAPDH Internal Reference Forward Primer (GAPDH-hDNA-F1): 5'-CCCCACACACATGCACT-3' (SEQ ID No: 16)

GAPDH Internal Reference Reverse Primer (GAPDH-hDNA-R1): 5'-CCTAGTCCCAGGGCTTTG-3' (SEQ ID No: 17)

GAPDH Internal Reference Probe (Q6-Gapdh-dnaP(Rox)): 5'-TGCCAAGTTGCCTGTCC-3' (SEQ ID No: 18)

Ⅳ. Preparation of the Reaction System

Preparation of the Primer and Probe Mixture: (taking 100 test portions as an example)

HPV: Dilute the primer and probe to a concentration of 100 μM. For HPV16, HPV18, and HPV33, 5 μl of each of the forward and reverse primers and 3 μl of the probe (250 nM/150 nM), totaling 39 μl; For the remaining primers and probes: Dilute the primer and probe to a concentration of 100 μM, 9 μl of each of the forward and reverse primers and 5 μl of the probe (450 nM/250 nM), totaling 56 μl; Blocker: Dilute the Blocker primer and probe to a concentration of 100 μM, take 1.25 μl (62.5 nM); Add 103.75 μl of water, totaling 200 μl, and add 2 μl to each reaction.

Preparation of the PCR Reaction System:

Component	Usage per Reaction
ddPCR Multiplex Supermix	5μL
Dithiothreitol (DTT)	1μL
DNA Template	8μL
Primer and Probe Mixture	2μL
Nuclease-Free Water	Up to 20μL

Ⅴ. Preparation of Droplets

Transfer the prepared 20 μl of the PCR reaction solution to the sample well of the droplet generation cartridge (DG8 cartridge), and then add 70 μl of the droplet generation oil to the oil well. Use the droplet generator of the QX200TM droplet digital PCR instrument to prepare the reaction droplets. Each droplet generation cartridge can complete the droplet preparation for 8 samples at the same time, which takes about 2.5 minutes.

Ⅵ. PCR Amplification

Transfer the droplets of each sample to the corresponding reaction wells of the 96-well

PCR reaction plate respectively. Seal it with an aluminum film by heat sealing (180°C, 5 sec), and then perform the amplification on a common PCR instrument. The PCR amplification of this experiment was completed on a Bio-Rad T100 PCR instrument, and the temperature program is shown in Table.

Table Cycling Protocol for Bio-Rad C100 thermal cycler

Temperature (℃)	Time	# Cycles
95	10min	1
94	30 sec.	50
58	1min	
98	10min	1

Check/adjust ramp rate settings to 2 ℃/sec. Use a hearted lid set to 105 ℃ and set the sample volume to 40 µl

Ⅶ. Detection and Data Analysis

Place the 96-well plate after PCR amplification into the droplet analyzer of the QX600TM droplet digital PCR instrument. The instrument automatically analyzes the fluorescence signals in each droplet of each sample. Then, the QX Manager Software automatically processes the data to obtain the copy number concentration (unit: copies/µl) of the target sequence in the PCR reaction system.
